# A molecular framework controlling style morphology in *Brassicaceae*

**DOI:** 10.1242/dev.158105

**Published:** 2018-03-01

**Authors:** Sara Simonini, Pauline Stephenson, Lars Østergaard

**Affiliations:** Crop Genetics Department, John Innes Centre, Norwich NR4 7UH, UK

**Keywords:** Transcription factors, Auxin, Style development, Gynoecium, *Brassicaceae*

## Abstract

Organ formation in multicellular organisms depends on the coordinated activities of regulatory components that integrate developmental and hormonal cues to control gene expression and mediate cell-type specification. For example, development of the *Arabidopsis* gynoecium is tightly controlled by distribution and synthesis of the plant hormone auxin. The functions of several transcription factors (TFs) have been linked with auxin dynamics during gynoecium development; yet how their activities are coordinated is not known. Here, we show that five such TFs function together to ensure polarity establishment at the gynoecium apex. The auxin response factor ETTIN (ARF3; herein, ETT) is a central component of this framework. Interaction of ETT with TF partners is sensitive to the presence of auxin and our results suggest that ETT forms part of a repressive gene-regulatory complex. We show that this function is conserved between members of the *Brassicaceae* family and that variation in an ETT subdomain affects interaction strengths and gynoecium morphology. These results suggest that variation in affinities between conserved TFs can lead to morphological differences and thus contribute to the evolution of diverse organ shapes.

## Abstract (precis)

**Summary:** Variation in interaction affinity between transcription factors of an ETTIN-containing complex underlies diversity of gynoecium style structure among members of the *Brassicacea* family.

## INTRODUCTION

**Fig. 1.**
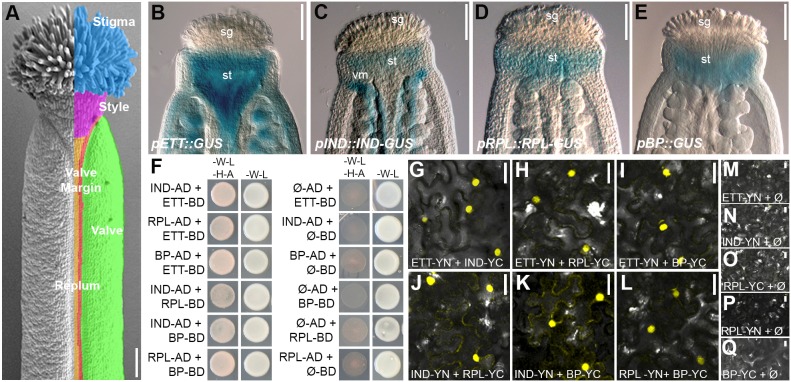
**Overlapping expression pattern of genes encoding ETT-interacting TFs.** (A) SEM image of wild-type mature gynoecium (stage 13). Tissues are colour-coded: cyan, stigma; pink, style; green, valves; red, valve margin; orange, replum. (B-E) GUS staining of *pETT(8kb)::GUS* (B), *pIND::IND-GUS* (C), *pRPL::RPL-GUS* (D) and *pBP::GUS* (E) marker lines showing overlapping expression patterns at the gynoecium apex at stage 11. (F) Y2H assay showing interactions between ETT, IND, RPL and BP (selective medium -W-L-H-A, left columns) and respective controls (permissive -W-L, right columns). (G-Q) BiFC assays between ETT, IND, RPL and BP in tobacco leaf epidermis cells (G-L) and respective controls (M-Q). sg, stigma; st, style; vm, valve margin. Scale bars: 100 µm in A-E, 20 µm in G-Q.

In the model plant *Arabidopsis thaliana*, the female reproductive organ, the gynoecium, develops at the centre of the flower. It is composed of an elongated ovary made up of two symmetrical valves separated by the replum and valve margin tissues and topped by a solid cylindrical style and stigmatic papillae (Fig. 1A). The formation of the gynoecium has been divided into discrete stages based on growth and appearance of specific tissues and developmental transitions (Smyth et al., 1990; Roeder and Yanofsky, 2006). The timely progression through these events relies on the interaction between transcription factor (TF) activities and hormone dynamics (Eklund et al., 2010; Girin et al., 2011; Heisler et al., 2001; Larsson et al., 2014; Moubayidin and Østergaard, 2014). Plant hormones such as auxin and cytokinin are intricately involved in the determination of gynoecium morphology and functionality (Nemhauser et al., 2000; Larsson et al., 2013; Reyes-Olalde et al., 2017; Zúñiga-Mayo et al., 2014). Plants exhibiting defects in synthesis, transport and/or perception of auxin typically show a dramatic reduction of fertility, mainly as a result of incorrect spatiotemporal development and differentiation of gynoecium tissues (Kuusk et al., 2002; Cheng et al., 2006; Stepanova et al., 2008; Trigueros et al., 2009; Moubayidin and Østergaard, 2014). Accumulation of auxin at the top of the young developing gynoecium is necessary to guide organ structure and differentiation of the style from the basal ovary (Moubayidin and Østergaard, 2014). This tightly controlled auxin distribution is ensured by the activity of the PIN-FORMED (PIN) auxin efflux carriers (Křeček et al., 2009; Trigueros et al., 2009), and plants exhibiting abnormal PIN distribution therefore develop styles with morphological defects (Moubayidin and Østergaard, 2014).

SPATULA (SPT) and INDEHISCENT (IND) are two bHLH-type TFs for which function in gynoecium development has been characterized in detail (Liljegren et al., 2004; Girin et al., 2011; Heisler et al., 2001; Moubayidin and Østergaard, 2014). SPT and IND proteins interact and together regulate the expression of downstream target genes at the gynoecium apex. This ensures that auxin becomes distributed in a ring surrounding the apex, which is required to create the radially symmetric style (Moubayidin and Østergaard, 2014). The auxin response factor ETTIN (ARF3; herein, ETT) is required for correct polarity establishment in the gynoecium (Sessions et al., 1997), including formation of correct style morphology (Sessions et al., 1997; Simonini et al., 2016). Indeed, *spt* and *ett* single mutants exhibit visible clefts or split styles at their gynoecium apex (Heisler et al., 2001; Simonini et al., 2016), henceforth referred to split-style defects, and double mutant combinations with *ind* further enhanced this defect (Girin et al., 2011; Moubayidin and Østergaard, 2014; Simonini et al., 2016).

In a recent study, we showed that IND and ETT proteins interact during style development (Simonini et al., 2016). This work also reported the interaction of ETT with other TFs, such as the homeodomain-containing proteins REPLUMLESS (RPL) (Roeder et al., 2003) and BREVIPEDICELLUS (BP/KNOTTED-LIKE ARABIDOPSIS THALIANA1) (Venglat et al., 2002). In addition to functions in stem development and phyllotaxis (Venglat et al., 2002; Byrne et al., 2003; Bencivenga et al., 2016), RPL and BP interact to ensure formation of the replum, which begins to differentiate during gynoecium development (Roeder and Yanofsky, 2006; Alonso-Cantabrana et al., 2007; González-Reig et al., 2012).

Here, we describe, through genetic and protein-protein interaction approaches, an additional route that gynoecium tissue identity factors, including ETT, IND, RPL and BP, adopt in order to ensure correct style morphogenesis and shape. Multiple mutant combinations between these factors lead to incorrect style development with severe split-style phenotypes. Furthermore, screening of mutant populations of *Brassica rapa*, a close relative of *Arabidopsis*, led to the identification of a subdomain in the ETT protein which is crucial for its heterodimerization. Specific amino acid substitutions in this subdomain correlate with style morphology defects and altered protein-protein dimerization sensitivity both in *Brassica* and *Arabidopsis*. In conclusion, our work describes a molecular framework that, through different levels of interaction affinities among a set of conserved TFs, is capable of generating different organ shapes and therefore likely contributes to the morphological diversity in reproductive structures observed in the *Brassicaceae* family.

## RESULTS

### Genes encoding four interacting TFs are expressed during style development

In order to identify additional factors that cooperate together with ETT and IND in style establishment, we previously screened the REGIA yeast-two-hybrid (Y2H) library of *Arabidopsis* TFs with ETT as bait (Paz-Ares and REGIA consortium, 2002; Simonini et al., 2016). Among the ETT interactors identified, the two HOMEOBOX TFs RPL (Roeder et al., 2003) and BP (Venglat et al., 2002) were particularly interesting because their expression pattern overlaps with ETT and IND at the stylar region of the developing gynoecium (Fig. 1B-E, Fig. S1), and because BP and RPL have previously been shown to interact and orchestrate common gynoecium developmental aspects, particularly the specification of the replum (Gonzáleg-Reig et al., 2012; Arnaud and Pautot, 2014). Interestingly, each of the four TFs (ETT, IND, BP and RPL) strongly interact with each other, both in the Y2H and in bifluorescence complementation (BiFC) assays (Fig. 1F-Q). Thus, these protein-protein affinities between ETT, IND, BP and RPL, added to their overlapping expression pattern and their involvement in regulating common target genes (Sorefan et al., 2009; Bencivenga et al., 2016; Simonini et al., 2016, 2017), might suggest a synergistic cooperation in controlling the expression of key factors to ultimately ensure robustness during the delicate stage of style formation.

### *ETT*, *IND*, *BP* and *RPL* genetically interact to specify correct style morphogenesis

**Fig. 2.**
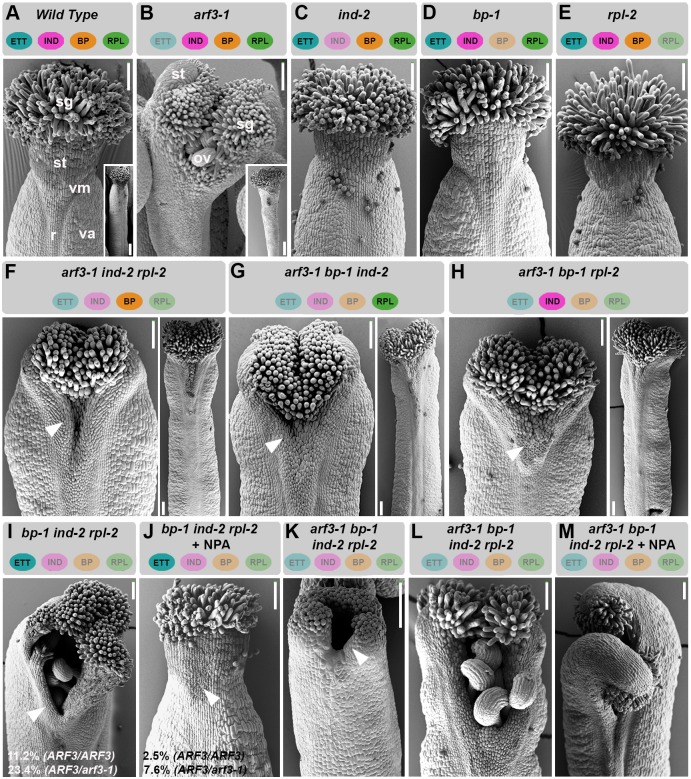
***ETT*, *BP*, *RPL* and *IND* genetically interact to ensure correct style formation.** (A-E) SEM images of gynoecium apices and whole carpel (insets) of wild type (A), and *arf3-1* (B), *ind-2* (C), *bp-1* (D) and *rpl-2* (E) single mutants. Gynoecium apices and whole carpel images correspond to two different gynoecia of the same genotype. (F-H) SEM images of gynoecium apices of *arf3-1 ind-2 rpl-2* (F), *arf3-1 bp-1 ind-2* (G) and *arf3-1 bp-1 rpl-2* (H) triple mutants and whole carpel (right). Gynoecium apices and whole carpel images correspond to two different gynoecia of the same genotype. Arrowheads point to regions of collapsed tissue at the gynoecium top. (I-J) SEM images of gynoecium apices of *ind-2 bp-1 rpl-2* triple mutant without (I) and with (J) NPA treatment. (K-M) SEM image of gynoecium apex of *arf3-1 ind-2 bp-1 rpl-2* quadruple mutant at stage 10 (K), at stage 12 (L) and at stage 12 after NPA treatment. Scale bars: 100 µm, 500 µm in insets. ov, ovules; r, replum; sg, stigma; st, style; va, valves; vm, valve margin.

To unravel the role of *ETT*, *IND*, *BP* and *RPL* during style development and a potential synergistic genetic interaction among them, the single mutants *arf3-1* (Okushima et al., 2005), *ind-2* (Liljegren et al., 2004), *rpl-2* (Roeder et al., 2003) and *bp-1* (Venglat et al., 2002), and all possible multiple combinations of these alleles in double, triple and quadruple mutants, were analysed and compared (Fig. 2, Fig. S2).

Among the single mutants, only *arf3-1*, which contains a T-DNA insertion in the *ETT* gene (Okushima et al., 2005), developed obvious defects in style morphology (Fig. 2A-E, Fig. S2A-E), accompanied by severe reduction of ovary length and width. The severity of defects in ovary development varies significantly among different *ett* alleles. By contrast, defects in style development are common to all the known *ett* alleles (Sessions et al., 1997; Nemhauser et al., 2000; Pekker et al., 2005; Xing et al., 2013; Simonini et al., 2016), suggesting that both null-alleles with no detectable *ETT* transcripts and truncated ETT variants derived from premature stop codons are defective in this process.

The defects at the level of style morphology of the *arf3-1* mutant were somewhat enhanced in double mutant combinations with *ind-2*, *bp-1* and *rpl-2* (Fig. S2F-K), but particularly the triple mutant combinations with *arf3-1* showed an enhanced defect in the style with areas of collapsed tissue (Fig. 2F-H, Fig. S2L-N). Interestingly, these triple mutant combinations showed a remarkable recovery of ovary morphology (Fig. 2B,F-H, Fig. S2B,L-N), suggesting that IND, BP and RPL impose tissue-identity constrains to the *arf3-1* ovary. In support of this interpretation, *IND*, *BP* and *RPL* were ectopically expressed in *arf3-1* mutant gynoecium (Fig. S2R-T), suggesting that ETT is also responsible for proper tissue identity specification through the regulation of key tissue-identity genes.

If ETT functions together with IND, BP and RPL in style formation, it is possible that mutations in all three interactors will lead to defective style formation. In agreement with this, 11.2% of the *ind-2 bp-1 rpl-2* triple mutant gynoecia analysed (*n*=120) developed clefts with variable degrees of depth, and this percentage was further increased to 23.4% when only one functional copy of *ETT* was present (*n*=120, triple mutant *ARF3/arf3-1 ind-2 bp-1 rpl-2*) (Fig. 2I, Fig. S2O). The lack of complete penetrance might be caused by additional ETT-interacting factors that have yet to be identified.

Treatment of *spt* mutant gynoecium with the auxin transport inhibitor 1-N-naphthylphthalamic acid (NPA) (Okada et al., 1991; Mattsson et al., 1999; Geldner et al., 2001) induces partial recovery of its split-style phenotype (Nemhauser et al., 2000). This result has been interpreted to mean that defects in auxin accumulation at the apex of *spt* mutant gynoecia is restored upon NPA treatment. By contrast, the *ett* mutant phenotype was not rescued by NPA (Nemhauser et al., 2000). NPA treatment was, however, able to rescue the split defect of *ARF3/ARF3 ind-2 bp-1 rpl-2* and *ARF3/arf3-1 ind-2 bp-1 rpl-2* mutants from 11.2% and 23.4% to 2.5% and 7.6%, respectively (*n*=120) (Fig. 2I,J). This genetic evidence confirms (for ETT and IND) and demonstrates (for BP and RPL) a clear function in style development connected with the control of auxin distribution at the gynoecium apex. NPA disturbs auxin distribution, possibly facilitating accumulation at the apical end of the gynoecium. Although this effect is sufficient to overcome the loss of *BP*, *IND* and *RPL*, which primarily function at the apex, the proposed role of *ETT* as an interpreter of cellular auxin levels is more widely required in the gynoecium ovary. This is in agreement with the observations described in Nemhauser et al. (2000).

Further corroboration for a role for all four TFs in the definition of style morphology came from the analysis of the quadruple *arf3-1 ind-2 bp-1 rpl-2* mutant (Fig. 2K,L). The defect is caused by a failure to fuse at the carpel margins, leading to a severe split-style phenotype with dramatic clefts extending approximately halfway down the ovary (Fig. 2L, Fig. S2P); these defects cannot be rescued by NPA treatment (Fig. 2M, Fig. S2Q). This is perhaps not an unexpected result because facilitating auxin accumulation at the gynoecium apex of the quadruple mutant cannot overcome the absence of ETT as interpreter of auxin cellular concentrations (Nemhauser et al., 2000: Simonini et al., 2016) and effector of auxin signalling (Simonini et al., 2016, 2017).

### *ETT*, *IND*, *BP* and *RPL* might have common target genes

The genetic and protein-protein interaction data presented above support a synergistic role for ETT, IND, BP and RPL in the developmental regulation of style morphology, and raise the possibility that these TFs regulate common target genes. To test this, we compared publicly available target datasets for ETT, IND, BP and RPL, and identified a number of potentially common targets. Among them, the gene encoding XYLOGLUCAN ENDOTRANSGLYCOSYLASE/HYDROLASE 7 (XTH7) emerged from the comparison of the target dataset of chromatin immunoprecipitation sequencing (ChIP-Seq) and transcriptome analyses of ETT and RPL (Bencivenga et al., 2016; Simonini et al., 2017), and a transcriptome profile of a dexametasone-inducible variant of IND (IND-GR) (Sorefan et al., 2009; Simonini et al., 2016). Interestingly, the *XTH7* promoter contains regulatory elements, which might provide binding sites for the four factors (Fig. S3A): an AuxRE element, the ETT preferential binding site (Franco-Zorrilla et al., 2014; Simonini et al., 2016); a RPL binding site (Bencivenga et al., 2016); a bHLH binding site (Sorefan et al., 2009; Girin et al., 2011); and an element proposed to be recognized by HOMEOBOX TFs such as BP (Viola and Gonzalez, 2006). In agreement with this, we found the *XTH7* gene to be misexpressed in inflorescence tissue from mutant combinations of *ETT*, *IND*, *RPL* and *BP* (Fig. S3B). The data show that *XTH7* mRNA is reduced in the *ett-3* mutant, suggesting that ETT is a positive regulator of *XTH7* expression. By contrast, BP, IND and RPL might function together as repressors of *XTH7*, because *XTH7* transcript levels are dramatically increased in the *bp-1 rpl-2 ind-2* triple mutant. This effect is reduced when *ett-3* is included in the quadruple mutant. These data therefore suggest that *XTH7* is a target for all TFs, but that they regulate expression of this gene in a differential manner.

### The molecular adaptor SEUSS regulates style formation

The molecular adaptor and transcriptional repressor SEUSS (SEU) has previously been proposed to regulate, in concert with ETT, several developmental responses in the gynoecium by repressing floral homeotic identity genes (Franks et al., 2002). Indeed, it has been proposed that ETT and SEU interact both genetically and at the protein level (Franks et al., 2002; Pfluger and Zambryski, 2004). Therefore, SEU might function together with ETT, IND, BP and RPL in the guidance of the developmental changes necessary for style differentiation.

**Fig. 3.**
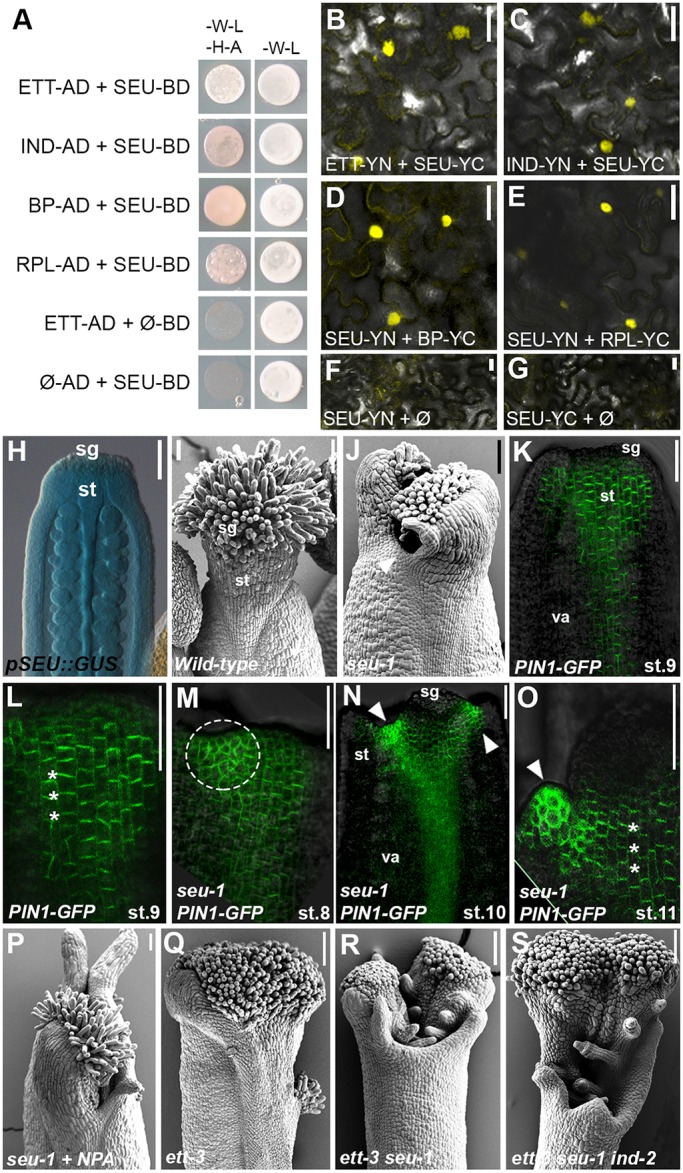
**SEU contributes to the regulation of style formation in concert with the ETT-IND-RPL-BP complex.** (A) Y2H assay between SEU and ETT, IND, BP and RPL (selective medium -W-L-H-A, left column) and respective controls (-W-L, right column). (B-G) BiFC assay in tobacco leaf epidermis cells, confirming the interaction between SEU and ETT (B), IND (C), BP (D) and RPL (E). F and G are negative controls. (H) GUS staining of *pSEU::GUS* marker line in wild-type gynoecium (stage 11). (I,J) SEM images of gynoecium apices of wild type (I) and *seu-1* (J) single mutant. (K-O) Confocal analysis of *pPIN1::PIN1-GFP* in wild type (K-L) and *seu-1* mutant (M-O) at indicated gynoecium stages. Asterisks indicate predominant PIN1 apical distribution; arrowheads indicate PIN1 apolar distribution. (P-S) SEM images of *seu-1* treated with NPA (P), *ett-3* (Q), *ett-3 seu-1* double mutant (R) and *ett-3 seu-1 ind-2* triple mutant (S). Scale bars: 20 µm in B-G, 100 µm in H-S. sg, stigma; st style; va, valves.

To test this hypothesis, we first analysed whether IND, BP and RPL could interact with SEU at the protein level. In parallel, to confirm the proposed ETT-SEU interaction (Pfluger and Zambryski, 2004), stable dimerization was detected between SEU-IND, SEU-BP and SEU-RPL dimers both in Y2H and BiFC assays (Fig. 3A-G). Moreover, analyses of the *pSEU::GUS* marker line (Gong et al., 2016) confirmed expression of *SEU* in the style region (Fig. 3H), coinciding with *ETT*, *IND*, *BP* and *RPL* expression in this tissue.

In agreement with previous reports on the role of SEU in style specification (Franks et al., 2002; Pfluger and Zambryski, 2004), *seu-1* mutant gynoecium exhibited a split-style phenotype (Fig. 3I,J, Fig. S4A). Because the split-style defect is closely connected with auxin distribution and abnormal cellular localization of the auxin efflux carrier PINs (Moubayidin and Østergaard, 2014), the localization of PIN1 was analysed and compared between the wild type and the *seu-1* mutant. During early stages of gynoecium development, PIN1-GFP localizes apically to promote acropetal auxin transport (from the base of the gynoecium to its top). Apolar distribution of PIN1-GFP at the gynoecium apex precedes differentiation of the style. This wild-type PIN1-GFP pattern (Fig. 3K,L) was altered in the *seu-1* mutant, with areas of the gynoecium apex presenting a mix of apolar and polar PIN1-GFP localization already from early developmental stages (Fig. 3M-O, Fig. S4B), thus suggesting erroneous auxin fluxes. These areas of apolar PIN1-GFP coincided with abnormalities at the *seu-1* gynoecium tops, ultimately leading to the *seu-1* split phenotype. Despite incorrect PIN1 localization in the *seu-1* mutant, the *seu-1* split style defect cannot be rescued by NPA treatment (Fig. 3P, Fig. S4C). This suggests that SEU has multiple functions in tissue specification and organ development in addition to the regulation of auxin transport.

The split-style defect in the *seu-1* mutant gynoecium was enhanced when combined with the *ett-3* mutant (Fig. 3Q,R, Fig. S4D) and dramatically exacerbated in the *ett-3 seu-1 ind-2* triple mutant (Fig. 3S, Fig. S4E,F). This result, combined with the finding that SEU and ETT have a number of target genes in common (Bao et al., 2010; Simonini et al., 2017), indicates that SEU is an additional factor that might contribute synergistically to the style formation together with ETT, IND, BP and RPL.

### Homologous functions of ETT, IND, BP, RPL and SEU in *Arabidopsis* and *Brassica*

**Fig. 4.**
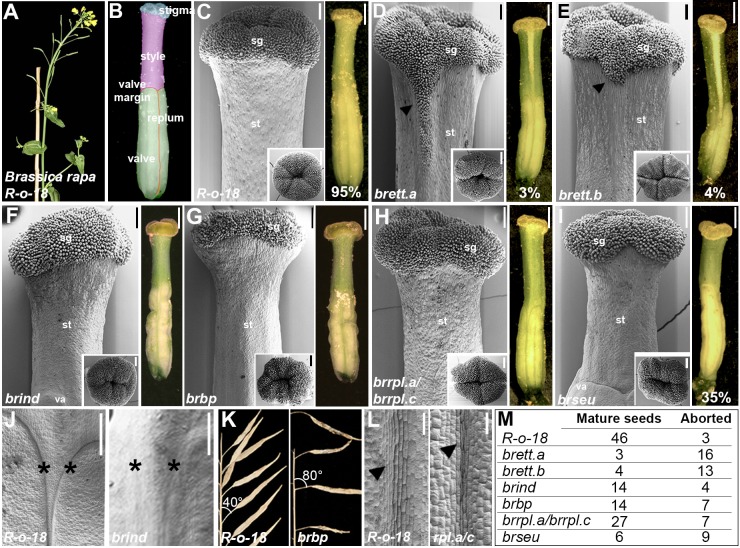
**ETT, IND, BP, RPL and SEU activity is conserved between *Arabidopsis* and *B. rapa*.** (A,B) *B. rapa* R-o-18 wild-type plant (A) and colour-coded gynoecium (B): cyan, stigma; pink, style; green, valves; red, valve margin; orange, replum. (C-I) SEM images of gynoecium tops (left) and optical microscopy images of whole gynoecium (right) of wild-type R-o-18 (C) and *brett.a* (D), *brett.b* (E), *brind* (F), *brbp* (G), *brrpl.a/brrpl.c* (H) and *brseu* (I) mutants. Insets are SEM images of apical view of gynoecium tops. (J) Valve-margin defects in *brind* mutant (right, asterisks) compared to wild-type R-o-18 (left, asterisks). (K) Pedicel defects with horizontal orientation of fruits in *brbp* mutant (right) compared to wild-type R-o-18 (left). (L) Replum defect in *brrpl.a/brrpl.c* double mutant (right, arrowhead), compared to wild-type R-o-18 (left, arrowhead). Scale bars: 200 µm in SEM images in C-I, 100 µm in SEM images in J and K, 1 mm in optical microscope images.

*Arabidopsis* belongs to the *Brassicaceae* family, which also includes members of the *Brassica* genus containing important crop plants such as oilseed rape (*Brassica napus*), turnip (*B. rapa*) (Fig. 4A) and broccoli (*Brassica* *oleracea* var. italica). Fruits from *Brassica* species are similar in architecture to *Arabidopsis* (Łangowski et al., 2016), with valves that are separated by valve margin and replum tissues (Fig. 4B), but in contrast to the short style in *Arabidopsis* fruits, styles from *Brassica* species are more elongated, making up a significant proportion of the entire fruit length (Fig. 4B). The *B. rapa* genome sequence (Wang et al., 2011) contains only a single copy of *IND*, *BP* and *SEU*, but two copies of *ETT* (Fig. S5) and three copies of *RPL*. Here, we will refer to these genes as *BrIND*, *BrBP*, *BrSEU*, *BrETT.a*, *BrETT.b*, *BrRPL.a*, *BrRPL.b* and *BrRPL.c*, respectively (full systematic names are listed in Table S1, according to Østergaard and King, 2008).

We first applied a genetics approach to assess the level of functional conservation of these five TFs between *Arabidopsis* and *Brassica*. To this end, we identified mutants from an EMS-induced mutant collection developed in the *B. rapa* accession R-o-18 (Stephenson et al., 2010). Mutations in *BrIND* and the *BrRPL* genes were previously identified (Girin et al., 2010; Stephenson et al., 2010), and by screening the exome-capture resource for this population (http://revgenuk.jic.ac.uk/), we also obtained mutations in the *BrBP*, *BrSEU*, *BrETT.a* and *BrETT.b* genes. The *brind* mutant allele containing a premature stop codon was already characterized for its strong indehiscent phenotype (described as *braA.ind.a-1* in Girin et al., 2010). Additionally, nonsense mutations were identified for *BrETT.a*, *BrETT.b*, *BrBP*, *BrRPL.a* and *BrSEU* loci (Fig. 4C-I, Fig. S5). Owing to the absence of nonsense mutations in the *BrRPL.b* and *BrRPL.c* genes, lines harbouring potentially disruptive missense mutations were isolated instead (Fig. S5).

*brett.a*, *brett.b*, *brind* and *brbp* single mutants, as well as the *brrpl.a*/*brrpl.c* double mutant, exhibited phenotypic defects in accordance with orthologous *Arabidopsis* mutants (*ett*, *ind*, *bp* and *rpl*) (Fig. 4C-L): overproliferation of stigmatic tissues with polarity defects at the gynoecium apex in both *brett.a* and *brett.b* mutants (Fig. 4C-E) (Sessions et al., 1997); lack of specification of the valve margin tissue in *brind* mutant fruits (Fig. 4C,F,J) (Liljegren et al., 2004); downwards orientation of flowers and fruits in *brbp* mutant (Fig. 4C,G,K) (Venglat et al., 2002); and narrow, poorly delineated replum in *brrpl.a*/*brrpl.c* mutant (Fig. 4C,H,L) (Roeder et al., 2003). Plants carrying a nonsense mutation in the first half of the *BrSEU* gene developed defects in ovary and style width and length (Fig. 4C,I), but only occasionally exhibited a split phenotype (Franks et al., 2002), suggesting either that alternative factors can compensate for the absence of *BrSEU* or that the truncated mutant version of the BrSEU protein retains partial functionality.

The loss-of-function phenotypes generally confirm a conserved function of these TFs between *Arabidopsis* and *B. rapa*. To test whether they also interact in a similar manner, we carried out Y2H assays. The results showed that BrETT.a, BrETT.b, BrIND, BrBP, BrRPL.a and BrSEU all interact with each other in a similar way to the homologous factors from *Arabidopsis* (Fig. S6), further supporting a high level of both functional and structural conservation within the *Brassicaceae* family.

### A single amino acid substitution in the ETT protein causes defects in style morphology

Twenty of the 23 auxin response factors (ARFs) in *Arabidopsis* contain a PB1 domain (Phom and Bem1) at their C-terminal half, which is required for responsiveness to auxin (Guilfoyle, 2015). ETT is a noncanonical ARF that instead of a PB1 domain possesses a unique domain with no sequence homology to any other known eukaryotic or prokaryotic protein. This domain is called the ETT-specific domain (ES domain) and is sufficient to mediate the reported auxin-sensitive interaction between ETT and its partners (Simonini et al., 2016).

**Fig. 5.**
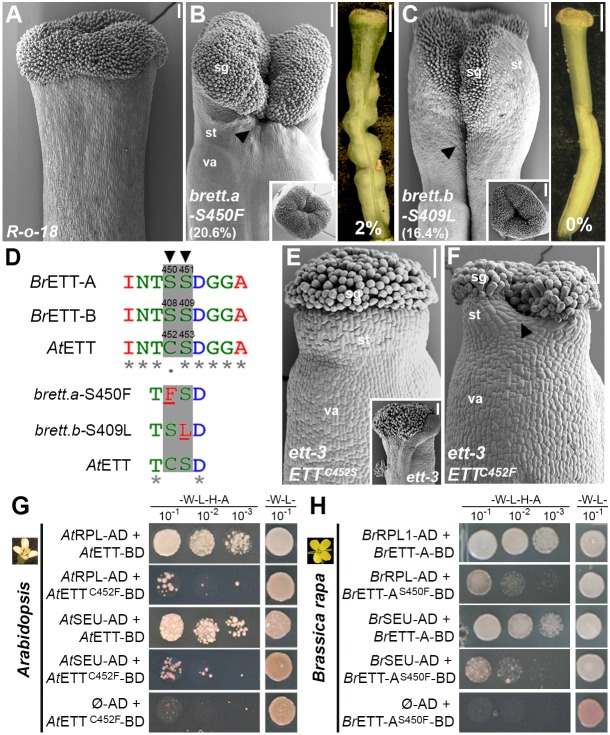
**A single amino acid substitution in ETT causes style defects and interferes with ETT heterodimerization.** (A-C) SEM images of gynoecium apices of R-o-18 (A), *brett.a_S450F* (B) and *brett.b_S409L* (C) mutants with severe split-style phenotypes (arrowheads) and reduced style length. Insets are SEM images of apical view of gynoecium tops and right panels in B and C are optical microscope images. (D) Protein sequence alignment by MUSCLE of BrETT.a, BrETT.b and AtETT, with focus on the region containing positions S450 (BrETT.a, arrowhead), S409 (BrETT.b, arrowhead) and C452, S453 (AtETT), and schematic representation of the corresponding S450F and S409L substitutions. (E) SEM images of *Arabidopsis* gynoecium apex of *ett-3* mutant plant (inset) fully complemented by the *pETT::ETT^C452S^* construct. (F) SEM images of *Arabidopsis* gynoecium apex of *ett-3* mutant plants transformed with the *pETT::ETT^C452F^* construct, showing a cleft (arrowhead) in the style. (G) Y2H assay between wild-type AtETT or AtETT^C452F^ variant with AtRPL and AtSEU. Interaction is compromized in combinations in which the AtETT^C452F^ variant is present. (H) Y2H assay between wild-type BrETT.a or BrETT.a^S450F^ variant with BrRPL.a and BrSEU. Similar to G, the strength of the interaction is compromized in combinations in which the BrETT.a^S450F^ variant is present. Scale bars: 100 µm in SEM images, 1 mm in optical microscope images. sg, stigma; st, style; va, valves.

With the aim to identify key amino acid residues that are important either for ETT dimerization with its partners or auxin sensitivity, we screened 10 *B. rapa* mutant lines from the exome-capture collection with missense mutations in the ES domain of *BrETT.a* or *BrETT.b* for defects at the gynoecium apex (Fig. S5). Among the 10 selected lines, JI3-3427a [serine-to-phenylalanine substitution at position 450 (S450F) of *BrETT.a*] and JI3-1096a [serine-to-leucine substitution at position 409 (S409L) of *BrETT.b*] developed gynoecia with styles of reduced length and with clefts at the apex (20.6% in the S450F line and 16.4% in the S409L line) (Fig. 5A-C).

The *BrETT.a*_S450 and the *BrETT.b*_S409 residues are located at the beginning of the ES domain and correspond respectively to cysteine 452 (C452) and serine 453 (S453) in AtETT (Fig. 5D, Fig. S5). Interestingly, C452 has previously been associated with auxin perception *in vivo* (Simonini et al., 2016), where the combination of C452S with a C506S mutation rendered ETT insensitive to auxin in its interaction with TF partners. The single C452S mutation in *Arabidopsis* ETT, which mimics the serine found at this position in BrETT.a was, however, not sufficient to alter ETT functionality or auxin perception (Simonini et al., 2016), and a *pETT::ETT^−C452S^* construct fully complemented the *ett-3* phenotype both in the ovary and in the stylar region (eight independent transformants) (Fig. 5E; Simonini et al., 2016).

Serine and cysteine are chemically very similar amino acids of almost identical size. By contrast, phenylalanine and leucine are larger and more hydrophobic, and the split-style phenotypes of *brett.a_S450F* and *brett.b_S409L* might therefore develop as a result of altered BrETT.a and BrETT.b activities. In support of this hypothesis, although *ett-3* mutant plants transformed with a *pETT::ETT^C452F^* construct showed full recovery of the ovary defects, they developed gynoecia with split styles (six independent transformants, with an average of 19.3% of the gynoecia analysed showing split style) (Fig. 5F, Fig. S8). These data therefore define an ETT subdomain, localized at the beginning of the ES domain and conserved among *Arabidopsis* and *Brassica*, that is required for its proper functionality (Fig. S7).

### Spilt-style defects correlate with ETT heterodimerization abilities

The penetrance of the split-style phenotypes in *brett.a_S450F*, *brett.b_S409L* and *pAtETT::AtETT^C452F^* is in the same range as the penetrance observed in the *ind-2 bp-1 rpl-2* triple mutant and the *ARF3/arf3-1 ind-2 bp-1 rpl-2* mutant background (11.2% and 23.4%, respectively) (Fig. 2I). We therefore wondered whether the dramatic amino acid substitutions at those positions affected ETT heterodimerization with its partners. In Y2H interaction assays, the strength of interactions between RPL and SEU with ETT variants AtETT^C452F^, AtETT^S453L^, BrETT.a^S450F^ and BrETT.b^S409L^ were significantly weaker compared with the combinations with wild-type ETT (Fig. 5G,H, Fig. S6). Additionally, because C452 in AtETT is involved in auxin perception (Simonini et al., 2016), the different Y2H interactions were also tested on medium supplemented with indole-3-acetic acid (IAA) as previously described (Simonini et al., 2016). Whereas interactions between wild-type AtETT and IND, RPL, BP and SEU are sensitive to IAA, dimers containing the ETT variants BrS450F, BrS409L, AtC452F and AtS453L were able to grow in the presence of IAA (Fig. S6). The effects of mutating these amino acids suggest that they have a rather direct role in the auxin sensing mechanism. This could either be through interaction with IAA or by facilitating a more indirect IAA-induced conformational change in the ETT protein structure.

In summary, this comparative analysis between *Arabidopsis* and *B. rapa* allowed us to establish that this subdomain of ETT proteins is important not only for auxin sensitivity as previously described (Simonini et al., 2016), but also for protein interactions. Altogether, the genetic and protein-protein interactions described in this work suggest that differential affinities between ETT, IND, BP, RPL and SEU factors lead to different style shapes in *Brassicaceae*.

## DISCUSSION

The different shapes observed among plant reproductive structures are genetically controlled by multiple players, which integrate developmental and hormonal cues in order to ensure successful reproduction. Multiple independent, yet interconnected, pathways are activated during gynoecium formation in order to properly differentiate and shape the various tissues, while providing robustness if one, or more, factors are malfunctioning.

The style and stigmatic structures that form at the apical part of the gynoecium are of fundamental importance for successful fertilization as they are responsible for pollen recognition, germination and subsequent guidance towards the ovules in the basal ovary. Several transcriptional regulators required for style development have been identified in *Arabidopsis*. These include bHLH proteins (SPT and IND), members of the SHI family [STYLISH1 (STY1) and STY2], NGATHA (NGA) proteins and ETT (Sessions et al., 1997; Heisler et al., 2001; Kuusk et al., 2002; Liljegren et al., 2004; Trigueros et al., 2009; Girin et al., 2011; Moubayidin and Østergaard, 2014; Simonini et al., 2016). Recently, a Y2H screen revealed auxin-sensitive interactions between ETT and proteins belonging to different TF families including IND and the HOMEOBOX TFs RPL and BP (Simonini et al., 2016). Because the expression patterns of *IND*, *RPL* and *BP* coincide with those of *ETT* in the style region (Simonini et al., 2016; Fig. 1B-E), we chose to study interactions between these four genes to uncover additional molecular frameworks that control style development. Expression of *RPL* and *BP* in the style region was previously detected (Ripoll et al., 2011), but no evidence for their role in the development of this structure has been proposed so far.

**Fig. 6.**
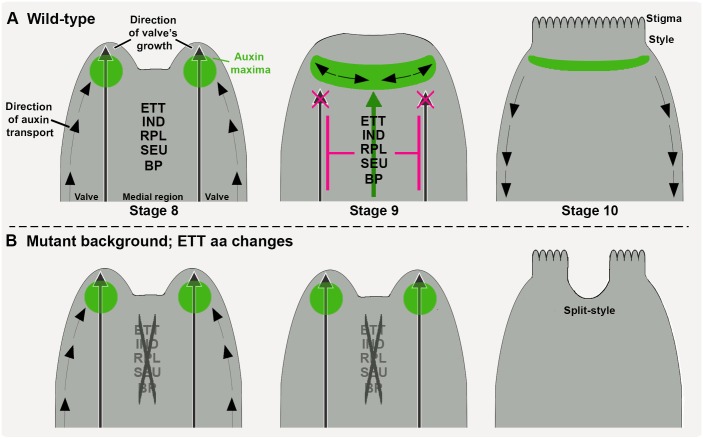
**Model for ETT-mediated gynoecium development via style type-specific TF interactions.** (A,B) Strong and stable protein-protein interactions between ETT, IND, RPL, BP and SEU promote correct style development and morphogenesis in a perfect cylindrical structure (A). In mutant lines in which amino-acid substitutions in ETT destabilize the strength of the interaction between ETT and SEU and RPL, development of split styles at the gynoecium apex can be observed (B). The progressive and dynamic distribution of auxin is indicated by polar transport (black arrows) and accumulation (green areas).

Genetic and protein-protein interactions between ETT, IND, BP, and RPL, and the severe split-style phenotypes exhibited by the *arf3-1 ind-2 bp-1 rpl-2* quadruple mutant (Fig. 2L), suggest that these TFs might regulate common target genes as dimers or higher-order complexes (Fig. 6). In support of this, comparison of publicly available target datasets for ETT, IND and RPL revealed shared common target genes among the four factors. One example is the *XTH7* gene. We found potential *cis* elements of all four TFs in the promoter region of *XTH7* and showed that *XTH7* is misexpressed in the mutant combinations analysed here (Fig. S3). These results therefore support that *XTH7* is a common target gene of all four TFs.

An additional factor that is able to interact both genetically and at the protein level with ETT, IND, BP and RPL is the co-adaptor SEU, the role of which in style development was previously proposed (Franks et al., 2002; Pfluger and Zambryski, 2004). Single *seu-1* mutants exhibit style defects that cannot be rescued by application of the auxin transport inhibitor NPA (Fig. 3M), suggesting that SEU has additional activities besides regulation of auxin fluxes. This result is in agreement with a previous observation that mutation in the Ser-Thr kinase *PINOID* (*PID*) gene enhances the gynoecium defects of the *seu-3* mutant (Pfluger and Zambryski, 2004). PID regulates cellular localization of PINs and consequently auxin distribution at the gynoecium apex (Benjamins et al., 2001; Moubayidin and Østergaard, 2014).

Although the proteins studied here can all interact in Y2H and BiFC, we cannot exclude the possibility that the phenotypic defects are caused by disrupting parallel genetic pathways. Since the phenotypic defects of multiple mutant combinations are gradually enhanced, it is, however, also possible that by progressively removing partners in a multimeric protein complex, such a complex will be increasingly compromised in regulating its target genes. It is for instance plausible that certain factors are required for the regulation of a subset of its targets, but not the entire target set. This concept is well established in the MADS-box field (Mateos et al., 2015), and future studies will be focused on analysing whether or not these factors function in one large complex or whether separate complexes form to ensure proper style development.

Analyses of lines carrying nonsense mutations in *ETT*, *IND*, *BP*, *RPL* and *SEU* loci in *B. rapa*, confirmed functional conservation for these factors in the *Brassicaceae* family (Fig. 4). Remarkably, single mutations in one of the two *B. rapa ETT* genes (*brett.a* and *brett.b)* were sufficient to induce development of phenotypical traits such as ectopic stigmatic tissue and gynoecium symmetry defects characteristic of *Arabidopsis ett* mutants (Fig. 4D,E). These data suggest either that both *BrETT.a* and *BrETT.b* genes are necessary for correct gynoecium development, or that expression of ETT-truncated peptides might have a negative effect. In this respect, it is worth noting that plants carrying nonsense mutations within the first half of the *ETT* gene, and hence expected to produce truncated ETT variants (e.g. *ett-1*, *arf3-1*), develop ovaries with very severe defects (Sessions et al., 1997; Nemhauser et al., 2000; Okushima et al., 2005; Pekker et al., 2005). In contrast to the ovary defects, all published *ett* alleles exhibit defects in style morphology, suggesting that full-length ETT protein is required during this process.

ETT encodes an atypical ARF that possesses a specific domain of unique sequence, called the ES domain, which is responsible for mediating protein-protein interaction and auxin sensitivity (Simonini et al., 2016). Through screening of missense mutations in *BrETT.a* and *BrETT.b* genes that correlate with split-style defects, the two ETT variants *brett.a_S450F* and *brett.b_S409L* were isolated (Fig. 5A-C). Both S-to-F and S-to-L substitutions, located at the beginning of the ES domain, constitute quite dramatic amino acid changes: serine is a small hydrophilic amino acid, which can reside both within the interior of a protein or on the protein surface. By contrast, phenylalanine and leucine are larger and more hydrophobic and hence better suited to being within protein hydrophobic cores. Thus, both S450F and S409L substitutions have the potential to alter the ETT structure and compromize its functionality.

The ES domain is responsible for mediating protein-protein interactions as well as auxin sensitivity (Simonini et al., 2016), and dramatic amino-acid substitutions at these positions affect both processes causing styles to be split (Fig. 5D). Interestingly, mutations in ETT that only affect auxin sensitivity, but maintain normal interaction with other proteins, was previously reported to cause overproliferation of stigmatic tissue, whereas split-style defects were never observed (Simonini et al., 2016). These results suggest that interactions between ETT and partners is important to first establish radial style formation, which precedes the formation of the auxin maxima at the gynoecium apex (Moubayidin and Østergaard, 2014). As the auxin maximum subsequently forms, this will lead to modulation of ETT-partner interactions for separate developmental responses.

Rather than abolishing interactions, the substitutions in BrETT^S450F^ (as in *brett.a_S450F*), AtETT^C452F^ (as in *pETT::ETT^C452F^* in *ett-3*) and BrETT^S409L^ (as in *brett.a_S409L*) affected predominantly the strength of the ETT-SEU and ETT-RPL dimerizations. Such reduced interaction strengths can alter target-gene regulation; indeed, the requirement of a protein partner for ETT to bind a set of target genes has been recently described (Simonini et al., 2017). Thus, weak affinities between ETT and protein partners might interfere with ETT recruitment to a set of genomic loci, which ultimately would lead to variation in target gene regulation. We therefore propose that style development in the *Brassicaceae* family is controlled by a conserved set of TFs, and that the diverse style morphologies have emerged owing to different interaction dynamics among them. Future studies will show whether similar variation in the strength of protein-protein interaction is responsible for diversity in the shape of other organs.

By combining available information and the knowledge acquired through this work, an integrated molecular mechanism for style establishment can be proposed, which takes into account the protein-protein interaction affinities between a set of conserved factors, their expression profile in relation to auxin dynamics, and the role that they have in regulating transcription (Fig. 6).

At early stages of gynoecium development, when style specification is about to take place, auxin starts to accumulate in lateral foci at the gynoecium apex, likely mediated through a combination of polar transport (Moubayidin and Østergaard, 2014; Larsson et al., 2014) and local biosynthesis controlled at least in part by the activities of STY1/2 and NGA3 (Eklund et al., 2010; Trigueros et al., 2009). At this stage, cell-fate reprogramming is pivotal to provide information for radial symmetry establishment and style differentiation at the top of the gynoecium (Moubayidin and Østergaard, 2014). Our data presented here suggest a model in which the transcriptional regulators ETT, IND, BP, RPL and SEU combine to control the transcription of genes responsible for style development and for auxin polar transport, in order to facilitate proper auxin distribution in this region. Both SEU and BP have been shown to interact with factors involved in chromatin modifications (Gonzalez et al., 2007; Zhao et al., 2015), and it is therefore possible that stable and durable repression is achieved through careful regulation of chromatin states at target gene loci. In plants that develop a split style (Fig. 6B), the protein-protein interactions among these factors are less stable, resulting in inefficient recruitment of the additional factors necessary for correct transcriptional modulation. Therefore, the different affinities between conserved interacting factors might result in the modulation of tissue growth and differentiation and thus contribute to the diversity of organ shapes that can be observed in nature.

## MATERIALS AND METHODS

### Plant materials and growth conditions

*Arabidopsis* plants were grown on soil in long-day conditions (16 h light/8 h dark). *B. rapa* plants were grown in soil in 1-l pots under long-day conditions (16 h light/8 h dark).

Mutant lines *ett-3*, *arf3-1*, *ind-2*, *bp-1*, *rpl-2* and *seu-1*, and the *pETT::ETT^C452S^* complementation line have previously been described (Sessions et al., 1997; Venglat et al., 2002; Franks et al., 2002; Roeder et al., 2003; Liljegren et al., 2004; Okushima et al., 2005; Simonini et al., 2016;). Marker lines *pETT::GUS*, *pSEU::GUS*, *pPIN1::PIN1-GFP*, *pIND::IND-GUS* and *pRPL::RPL-GUS* have previously been described (Benková et al., 2003; Roeder et al., 2003; Ng et al., 2009; Girin et al., 2011; Gong et al., 2016). *pBP::GUS* is available at the Nottingham Arabidopsis Sock Centre (NASC stock number N6141).

The *B. rapa brind.a*, *brrpl.a* and *brrpl.c* mutants were previously described (Stephenson et al., 2010); the *brett.a*, *brett.b*, *brett.a_S450F*, *brett.b_S409L*, *brbp*, and *brseu* were obtained by searching the 1154 *B. rapa* mutant lines sequenced by exome capture from the RevGenUK reverse genetics platform at the John Innes Centre (https://www.jic.ac.uk/technologies/genomic-services/revgenuk-tilling-reverse-genetics/).

### GUS assays and scanning electron microscopy

GUS assay and scanning electron microscopy (SEM) were performed as previously described (Moubayidin and Østergaard, 2014).

### *In planta* vector construction

For *pETT::ETT^C452F^* line, the C452F mutation was introduced to the *pETT::ETT^WT^* construct (Simonini et al., 2016) by site-specific mutagenesis using the Q5^®^ Site-Directed Mutagenesis Kit (NEB). Vector was transformed in *Agrobacterium tumefaciens* GV3101 and *ett-3* mutant plants were transformed following the floral-dip method (Clough and Bent, 1998). Quantitative real-time PCR analysis was used to estimate the numbers of transgene copies in the 24 individual transgenic lines obtained, similar to the approach previously reported in Bartlett et al. (2008). Only lines harbouring one, two or three transgene copies were considered for the phenotypical analyses, as lines with four or more copies of the transgene showed *ett*-related phenotypes of severity correlated with the number of insertions.

### Y2H and BiFC assays

The Y2H assays were performed at 28°C in the yeast strain AH109 (Clontech) using the co-transformation technique. Coding sequences were cloned into Gateway vector GAL4 system (pGADT7 and pGBKT7; Clontech) passing through pDONR207 (Life Technologies). Strength of interaction was tested on selective yeast synthetic dropout medium (YSD) lacking Leu (L), Trp (W), Adenine (A), and His (H), supplemented with different concentrations of 3-aminotriazole (Sigma-Aldrich). IAA (Sigma-Aldrich) was dissolved in ethanol and added at the desired concentration directly to the cooling media.

BiFC was performed as previously described (Belda-Palazon et al., 2012). Open-reading frames of full length selected genes were cloned in the pYFPN43 and pYFPC43 (http://www.ibmcp.upv.es/FerrandoLabVectors.php), passing through pDONR207 (Life Technologies). pEAQ-HT vector (Sainsbury et al., 2009), which expresses the silencing suppressor p19 of tomato bushy stunt virus, was added to the vector combinations. Images were taken 72 h after infiltration.

### Expression analysis

Three samples of few inflorescences each were harvested for each genotype, and RNA was extracted with the Qiagen Plant RNeasy extraction kit. cDNA was synthetized with M-MMV reverse transcriptase (Invitrogen). Real-Time PCR was performed in technical quadruplicate using a Bio-Rad CFX96 Real-Time System and SYBR Green JumpStart Taq ReadyMix (Sigma-Aldrich). Primers used are listed in Table S2.

## Supplementary Material

Supplementary information

Supplementary information

## References

[DEV158105C1] Alonso-Cantabrana H., Ripoll J. J., Ochando I., Vera A., Ferrándiz C. and Martínez-Laborda A. (2007). Common regulatory networks in leaf and fruit patterning revealed by mutations in the Arabidopsis ASYMMETRIC LEAVES1 gene. *Development* 134, 2663-2671. 10.1242/dev.02864 17592013

[DEV158105C2] Arnaud N. and Pautot V. (2014). Ring the BELL and tie the KNOX: roles for TALEs in gynoecium development. *Front. Plant Sci.* 5, 93 10.3389/fpls.2014.00093 24688486 PMC3960571

[DEV158105C3] Bao F., Azhakanandam S. and Franks R. G. (2010). SEUSS and SEUSS-LIKE transcriptional adaptors regulate floral and embryonic development in Arabidopsis. *Plant Physiol.* 152, 821-836. 10.1104/pp.109.146183 20007451 PMC2815852

[DEV158105C4] Bartlett J. G., Alves S. C., Smedley M., Snape J. W. and Harwood W. A. (2008). High-throughput Agrobacterium-mediated barley transformation. *Plant Methods* 4, 22 10.1186/1746-4811-4-22 18822125 PMC2562381

[DEV158105C5] Belda-Palazon B., Ruiz L., Martí E., Tárraga S., Tiburcio A. F., Culiáñez F., Farràs R., Carrasco P. and Ferrando A. (2012). Aminopropyltransferases involved in polyamine biosynthesis localize preferentially in the nucleus of plant cells. *PLoS ONE* 7, e46907 10.1371/journal.pone.0046907 23056524 PMC3466176

[DEV158105C6] Bencivenga S., Serrano-Mislata A., Bush M., Fox S. and Sablowski R. (2016). Control of oriented tissue growth through repression of organ boundary genes promotes stem morphogenesis. *Dev. Cell* 39, 198-208. 10.1016/j.devcel.2016.08.013 27666746 PMC5084710

[DEV158105C7] Benjamins R., Quint A., Weijers D., Hooykaas P. and Offringa R. (2001). The PINOID protein kinase regulates organ development in *Arabidopsis* by enhancing polar auxin transport. *Development* 128, 4057-4067. 11641228 10.1242/dev.128.20.4057

[DEV158105C8] Benková E., Michniewicz M., Sauer M., Teichmann T., Seifertová D., Jürgens G. and Friml J. (2003). Local, efflux-dependent auxin gradients as a common module for plant organ formation. *Cell* 115, 591-602. 10.1016/S0092-8674(03)00924-3 14651850

[DEV158105C9] Byrne M. E., Groover A. T., Fontana J. R. and Martienssen R. A. (2003). Phyllotactic pattern and stem cell fate are determined by the Arabidopsis homeobox gene BELLRINGER. *Development* 130, 3941-3950. 10.1242/dev.00620 12874117

[DEV158105C10] Cheng Y., Dai X. and Zhao Y. (2006). Auxin biosynthesis by the YUCCA Flavin monooxygenases controls the formation of floral organs and vascular tissues in Arabidopsis. *Genes Dev.* 20, 1790-1799. 10.1101/gad.1415106 16818609 PMC1522075

[DEV158105C11] Clough S. J. and Bent A. F. (1998). Floral dip: a simplified method for Agrobacterium-mediated transformation of Arabidopsis thaliana. *Plant J.* 16, 735-743. 10.1046/j.1365-313x.1998.00343.x 10069079

[DEV158105C12] Eklund D. M., Ståldal V., Valsecchi I., Cierlik I., Eriksson C., Hiratsu K., Ohme-Takagi M., Sundstrom J. F., Thelander M., Ezcurra I. et al. (2010). The Arabidopsis thaliana STYLISH1 protein acts as a transcriptional activator regulating auxin biosynthesis. *Plant Cell* 22, 349-363. 10.1105/tpc.108.064816 20154152 PMC2845406

[DEV158105C13] Franco-Zorrilla J. M., López-Vidriero I., Carrasco J. L., Godoy M., Vera P. and Solano R. (2014). DNA-binding specificities of plant transcription factors and their potential to define target genes. *Proc. Natl. Acad. Sci. USA* 111, 2367-2372. 10.1073/pnas.1316278111 24477691 PMC3926073

[DEV158105C14] Franks R. G., Wang C., Levin J. Z. and Liu Z. (2002). SEUSS, a member of a novel family of plant regulatory proteins, represses floral homeotic gene expression with LEUNIG. *Development* 129, 253-263. 11782418 10.1242/dev.129.1.253

[DEV158105C15] Geldner N., Friml J., Stierhof Y.-D., Jürgens G. and Palme K. (2001). Auxin transport inhibitors block PIN1 cycling and vesicle trafficking. *Nature* 413, 425-428. 10.1038/35096571 11574889

[DEV158105C16] Girin T., Stephenson P., Goldsack C. M. P., Perez A., Pires N., Sparrow P. A., Wood T. A., Yanofsky M. F. and Østergaard L. (2010). *Brassicaceae INDEHISCENT* genes specify valve margin cell fate and repress replum formation. *Plant J.* 63, 329-338. 10.1111/j.1365-313X.2010.04244.x 20444234

[DEV158105C17] Girin T., Paicu T., Stephenson P., Fuentes S., Körner E., O'Brien M., Sorefan K., Wood T. A., Balanza V., Ferrandiz C. et al. (2011). INDEHISCENT and SPATULA interact to specify carpel and valve margin tissue and thus promote seed dispersal in *Arabidopsis*. *Plant Cell* 23, 3641-3653. 10.1105/tpc.111.090944 21990939 PMC3229140

[DEV158105C18] Gong X., Flores-Vergara M. A., Hong J. H., Chu H., Lim J., Franks R. G., Liu Z. and Xu J. (2016). SEUSS integrates gibberellin signaling with transcriptional inputs from the SHR-SCR-SCL3 module to regulate middle cortex formation in the arabidopsis root. *Plant Physiol.* 170, 1675-1683. 10.1104/pp.15.01501 26818732 PMC4775121

[DEV158105C19] Gonzalez D., Bowen A. J., Carroll T. S. and Conlan R. S. (2007). The transcription corepressor LEUNIG interacts with the histone deacetylase HDA19 and mediator components MED14 (SWP) and CDK8 (HEN3) to repress transcription. *Mol. Cell. Biol.* 27, 5306-5315. 10.1128/MCB.01912-06 17526732 PMC1952085

[DEV158105C20] González-Reig S., Ripoll J. J., Vera A., Yanofsky M. F. and Martínez-Laborda A. (2012). Antagonistic gene activities determine the formation of pattern elements along the mediolateral axis of the Arabidopsis fruit. *PLoS Genet.* 8, e1003020 10.1371/journal.pgen.1003020 23133401 PMC3486860

[DEV158105C21] Guilfoyle T. J. (2015). The PB1 domain in AUxin response factor and Aux/IAA proteins: a versatile Protein interaction module in auxin response. *Plant Cell* 27, 33-43. 10.1105/tpc.114.132753 25604444 PMC4330575

[DEV158105C22] Heisler M., Atkinson A., Bylstra Y., Walsh R. and Smyth D. R. (2001). *SPATULA*, a gene that controls development of carpel margin tissues in *Arabidopsis*, encodes a bHLH protein. *Development* 128, 1089-1098. 11245574 10.1242/dev.128.7.1089

[DEV158105C23] Křeček P., Skůpa P., Libus J., Naramoto S., Tejos R., Friml J. and Zažímalová E. (2009). The PIN-FORMED (PIN) protein family of auxin transporters. *Genome Biol.* 10, 249 10.1186/gb-2009-10-12-249 20053306 PMC2812941

[DEV158105C24] Kuusk S., Sohlberg J. J., Long J. A., Fridborg I. and Sundberg E. (2002). STY1 and STY2 promote the formation of apical tissues during Arabidopsis gynoecium development. *Development* 129, 4707-4717. 12361963 10.1242/dev.129.20.4707

[DEV158105C25] Łangowski Ł., Stacey N. and Østergaard L. (2016). Diversification of fruit shape in the Brassicaceae family. *Plant Reprod.* 29, 149-163. 10.1007/s00497-016-0278-6 27016361

[DEV158105C26] Larsson E., Franks R. G. and Sundberg E. (2013). Auxin and the Arabidopsis thaliana gynoecium. *J. Exp. Bot.* 64, 2619-2627. 10.1093/jxb/ert099 23585670

[DEV158105C27] Larsson E., Roberts C. J., Claes A. R., Franks R. G. and Sundberg E. (2014). Polar auxin transport is essential for medial versus lateral tissue specification and vascular-mediated valve outgrowth in Arabidopsis gynoecia. *Plant Physiol.* 166, 1998-2012. 10.1104/pp.114.245951 25332506 PMC4256862

[DEV158105C28] Liljegren S. J., Roeder A. H. K., Kempin S. A., Gremski K., Østergaard L., Guimil S., Reyes D. K. and Yanofsky M. F. (2004). Control of fruit patterning in *Arabidopsis* by *INDEHISCENT*. *Cell* 116, 843-853. 10.1016/S0092-8674(04)00217-X 15035986

[DEV158105C29] Mateos J. L., Madrigal P., Tsuda K., Rawat V., Richter R., Romera-Branchat M., Fornara F., Schneeberger K., Krajewski P. and Coupland G. (2015). Combinatorial activities of SHORT VEGETATIVE PHASE and FLOWERING LOCUS C define distinct modes of flowering regulation in *Arabidopsis*. *Genome Biol.* 16:31 10.1186/s13059-015-0597-1 25853185 PMC4378019

[DEV158105C30] Mattsson J., Sung Z. R. and Berleth T. (1999). Responses of plant vascular systems to auxin transport inhibition. *Development* 126, 2979-2791. 10.1242/dev.126.13.2979 10357941

[DEV158105C31] Moubayidin L. and Østergaard L. (2014). Dynamic control of auxin distribution imposes a bilateral-to-radial symmetry switch during gynoecium development. *Curr. Biol.* 24, 2743-2748. 10.1016/j.cub.2014.09.080 25455035 PMC4245708

[DEV158105C32] Nemhauser J., Feldmann L. J. and Zambryski P. C. (2000). Auxin and ETTIN in *Arabidopsis* gynoecium morphogenesis. *Development* 127, 3877-3888. 10952886 10.1242/dev.127.18.3877

[DEV158105C33] Ng K.-H., Yu H. and Ito T. (2009). AGAMOUS controls GIANT KILLER, a multifunctional chromatin modifier in reproductive organ patterning and differentiation. *PLoS Biol.* 7, e1000251 10.1371/journal.pbio.1000251 19956801 PMC2774341

[DEV158105C34] Okada K., Ueda J., Komaki M. K., Bell C. J. and Shimura Y. (1991). Requirement of the auxin polar transport system in early stages of arabidopsis floral bud formation. *Plant Cell* 3, 677-684. 10.1105/tpc.3.7.677 12324609 PMC160035

[DEV158105C35] Okushima Y., Overvoorde P. J., Arima K., Alonso J. M., Chan A., Chang C., Ecker J. R., Hughes B., Lui A., Nguyen D. et al. (2005). Functional genomic analysis of the AUXIN RESPONSE FACTOR gene family members in Arabidopsis thaliana: unique and overlapping functions of ARF7 and ARF19. *Plant Cell* 17, 444-463. 10.1105/tpc.104.028316 15659631 PMC548818

[DEV158105C36] Østergaard L. and King G. J. (2008). Standardized gene nomenclature for the Brassica genus. *Plant Methods* 4, 10 10.1186/1746-4811-4-10 18492252 PMC2408569

[DEV158105C37] Paz-Ares J. and Regia Consortium (2002). REGIA, an EU project on functional genomics of transcription factors from Arabidopsis thaliana. *Comp. Funct. Genomics* 3, 102-108. 10.1002/cfg.146 18628849 PMC2447270

[DEV158105C38] Pekker I., Alvarez J. P. and Eshed Y. (2005). Auxin response factors mediate Arabidopsis organ asymmetry via modulation of KANADI activity. *Plant Cell* 17, 2899-2910. 10.1105/tpc.105.034876 16199616 PMC1276018

[DEV158105C39] Pfluger J. and Zambryski P. (2004). The role of SEUSS in auxin response and floral organ patterning. *Development* 131, 4697-4707. 10.1242/dev.01306 15358669

[DEV158105C40] Reyes-Olalde J. I., Zúñiga-Mayo V. M., Serwatowska J., Chavez Montes R. A., Lozano-Sotomayor P., Herrera-Ubaldo H., Gonzalez-Aguilera K. L., Ballester P., Ripoll J. J., Ezquer I. et al. (2017). The bHLH transcription factor SPATULA enable cytokinin signaling, and both activate auxin biosynthesis and transport genes at the medial domain of the gynoecium. *PLoS Genet.* 13, e1006726 10.1371/journal.pgen.1006726 28388635 PMC5400277

[DEV158105C41] Ripoll J. J., Roeder A. H. K., Ditta G. S. and Yanofsky M. F. (2011). A novel role for the floral homeotic gene APETALA2 during Arabidopsis fruit development. *Development* 138, 5167-5176. 10.1242/dev.073031 22031547

[DEV158105C42] Roeder A. H. K. and Yanofsky M. F. (2006). Fruit development in Arabidopsis. *Arabidopsis Book* 4, e0075 10.1199/tab.0075 22303227 PMC3243326

[DEV158105C43] Roeder A. H. K., Ferrándiz C. and Yanofsky M. F. (2003). The role of the REPLUMLESS homeodomain protein in patterning the *Arabidopsis* fruit. *Curr. Biol.* 13, 1630-1635. 10.1016/j.cub.2003.08.027 13678595

[DEV158105C44] Sainsbury F., Thuenemann E. C. and Lomonossoff G. P. (2009). pEAQ: versatile expression vectors for easy and quick transient expression of heterologous proteins in plants. *Plant Biotechnol. J.* 7, 682-693. 10.1111/j.1467-7652.2009.00434.x 19627561

[DEV158105C45] Sessions A., Nemhauser J. L., McColl A., Roe J. L., Feldmann K. A. and Zambryski P. C. (1997). ETTIN patterns the Arabidopsis floral meristem and reproductive organs. *Development* 124, 4481-4491. 9409666 10.1242/dev.124.22.4481

[DEV158105C46] Simonini S., Deb J., Moubayidin L., Stephenson P., Valluru M., Freire-Rios A., Sorefan K., Weijers D., Friml J. and Østergaard L. (2016). A noncanonical auxin-sensing mechanism is required for organ morphogenesis in Arabidopsis. *Genes Dev.* 30, 2286-2296. 10.1101/gad.285361.116 27898393 PMC5110995

[DEV158105C47] Simonini S., Bencivenga S., Trick M. and Østergaard L. (2017). Auxin-induced modulation of ETTIN activity orchestrates gene expression in Arabidopsis. *Plant Cell* 29, 1864-1882. 10.1105/tpc.17.00389 28804059 PMC5590509

[DEV158105C48] Smyth D. R., Bowman J. L. and Meyerowitz E. M. (1990). Early flower development in *Arabidopsis*. *Plant Cell* 2, 755-767. 10.1105/tpc.2.8.755 2152125 PMC159928

[DEV158105C49] Sorefan K., Girin T., Liljegren S. J., Ljung K., Robles P., Galván-Ampudia C. S., Offringa R., Friml J., Yanofsky M. F. and Østergaard L. (2009). A regulated auxin minimum is required for seed dispersal in Arabidopsis. *Nature* 459, 583-586. 10.1038/nature07875 19478783

[DEV158105C50] Stepanova A. N., Robertson-Hoyt J., Yun J., Benavente L. M., Xie D.-Y., Doležal K., Schlereth A., Jürgens G. and Alonso J. M. (2008). TAA1-mediated auxin biosynthesis is essential for hormone crosstalk and plant development. *Cell* 133, 177-191. 10.1016/j.cell.2008.01.047 18394997

[DEV158105C51] Stephenson P., Baker D., Girin T., Perez A., Amoah S., King G. J. and Østergaard, L. (2010). A rich TILLING resource for studying gene function in *Brassica rapa*. *BMC Plant Biol.* 10, 62 10.1186/1471-2229-10-62 20380715 PMC2923536

[DEV158105C52] Trigueros M., Navarrete-Gómez M., Sato S., Christensen S. K., Pelaz S., Weigel D., Yanofsky M. F. and Ferrandiz C. (2009). The NGATHA genes direct style development in the Arabidopsis gynoecium. *Plant Cell* 21, 1394-1409. 10.1105/tpc.109.065508 19435937 PMC2700528

[DEV158105C53] Venglat S. P., Dumonceaux T., Rozwadowski K., Parnell L., Babic V., Keller W., Martienssen R., Selvaraj G. and Datla R. (2002). The homeobox gene BREVIPEDICELLUS is a key regulator of inflorescence architecture in Arabidopsis. *Proc. Natl. Acad. Sci. USA* 99, 4730-4735. 10.1073/pnas.072626099 11917137 PMC123716

[DEV158105C54] Viola I. L. and Gonzalez D. H. (2006). Interaction of the BELL-like protein ATH1 with DNA: role of homeodomain residue 54 in specifying the different binding properties of BELL and KNOX proteins. *Biol. Chem.* 387, 31-40. 10.1515/BC.2006.006 16497162

[DEV158105C55] Wang X., Wang H., Wang J., Sun R., Wu J., Liu S., Bai Y., Mun J.-H., Bancroft I., Cheng F. et al. (2011). The genome of the mesopolyploid crop species *Brassica rapa*. *Nat. Genet.* 43, 1035-1039. 10.1038/ng.919 21873998

[DEV158105C56] Xing S., Salinas M., Garcia-Molina A., Höhmann S., Berndtgen R. and Huijser P. (2013). *SPL8* and mIR156-targeted *SPL* genes redundantly regulate Arabidopsis gynoecium differential patterning. *Plant J.* 75, 566-577. 10.1111/tpj.12221 23621152

[DEV158105C57] Zhao M., Yang S., Chen C. Y., Li C., Shan W., Lu W., Wu K. and Chen X. (2015). Arabidopsis BREVIPEDICELLUS interacts with the SWI2/SNF2 chromatin remodeling ATPase BRAHMA to regulate KNAT2 and KNAT6 expression in control of inflorescence architecture. *PLoS Genet.* 11, e1005125 10.1371/journal.pgen.1005125 25822547 PMC4379049

[DEV158105C58] Zúñiga-Mayo V. M., Reyes-Olalde J. I., Marsch-Martinez N. and de Folter S. (2014). Cytokinin treatments affect the apical-basal patterning of the Arabidopsis gynoecium and resemble the effects of polar auxin transport inhibition. *Front. Plant Sci.* 5, 191 10.3389/fpls.2014.00191 24860582 PMC4030163

