# Supplementary material for: A molecular framework controlling style morphology in Brassicaceae
Source: Development. 2018 Mar 1;145(5):dev158105. doi: 10.1242/dev.158105 (PMC5868994; doi:10.1242/dev.158105)
Supplement: Supplementary information [file develop-145-158105-s1.pdf]

Table S1. Nomenclature for *B. rapa* genes.

ETT	*BraA.ETT.a, BraA.ETT.b*
IND	*BraA.IND.a*
RPL	*BraA.RPL.a, BraA.RPL.b, BraA.RPL.c*
BP	*BraA.BP.a*
SEU	*BraA.SEU.a*

Table S2. Primers used in this manuscript.

Arabidopsis ETT for Y2H and BiFC	ggggacaagtttgtacaaaaaagcaggcttcATGGGTGGTTTAATCGATCTG<br>GGGGACCACTTTGTACAAGAAAGCTGGGTGCTAGAGAGCAATGTCTAGCA
Arabidopsis RPL for Y2H and BiFC	ggggacaagtttgtacaaaaaagcaggcttcATGGCTGATGCATACGAGCC<br>GGGGACCACTTTGTACAAGAAAGCTGGGTCTCAACCTACAAAATCATGTAGAAAC
Arabidopsis BP for Y2H and BiFC	ggggacaagtttgtacaaaaaagcaggcttcATGAAATATAAATCGAGATTAAATAG<br>GGGGACCACTTTGTACAAGAAAGCTGGGTGTTATGGACCGAGACGATAAGGTCCATC
Arabidopsis IND for Y2H and BiFC	ggggacaagtttgtacaaaaaagcaggcttcATGATGGAGCCTCAGCCTCACC<br>GGGGACCACTTTGTACAAGAAAGCTGGGTGTCAGGGTTGGGAGTTGTGGT
Arabidopsis SEU for Y2H and BiFC	ggggacaagtttgtacaaaaaagcaggcttcATGGTACCATCAGAGCCGCCTAATCC<br>GGGGACCACTTTGTACAAGAAAGCTGGGTGATGGTACCATCAGAGCCGCCTAATCC
B.rapa ETT.a for Y2H	ggggacaagtttgtacaaaaaagcaggcttcatgggtggtttaattgatctgaacg<br>GGGGACCACTTTGTACAAGAAAGCTGGGTGctagagagcaatgtctagcaacatatc
B.rapa ETT.b for Y2H	ggggacaagtttgtacaaaaaagcaggcttcATGGGTGGCTTAATCGATCTG<br>GGGGACCACTTTGTACAAGAAAGCTGGGTCCTAGAGAGCAATGTCTAGCAAC
B.rapa RPL.a for Y2H	ggggacaagtttgtacaaaaaagcaggcttcATGGCCGATGCTTACGAGCC<br>GGGGACCACTTTGTACAAGAAAGCTGGGTCTCAACCTACAAAATCATGTAGAAAC
B.rapa BP for Y2H	ggggacaagtttgtacaaaaaagcaggcttcATGGAAGAATATCAACATGAAAGC<br>GGGGACCACTTTGTACAAGAAAGCTGGGTGTTATGGTCCAAGACGATAAGGGCC
B.rapa IND for Y2H	ggggacaagtttgtacaaaaaagcaggcttcATGTCTGGCTCAAAAGCAGATGC<br>GGGGACCACTTTGTACAAGAAAGCTGGGTGTCAGACATAGGAGCTCCAAGCTG
B.rapa SEU for Y2H	ggggacaagtttgtacaaaaaagcaggcttcatggtgccatcagaggcgccaaatcc<br>GGGGACCACTTTGTACAAGAAAGCTGGGTGtcatgcgttccaatcacgctgaaaattg
Arabidopsis UBQ10 for QTRPCR	AGAACTCTTGCTGACTACAATATCCAG<br>ATAGTTTTCCCAGTCAACGTCTTAAC
Arabidopsis XTH7 for QTRPCR	ATTGACGGAGGCAGAGCCATTC<br>TCTTCGAAGCAAATCCACATCCTG

Development • Supplementary information

S1 Figure.

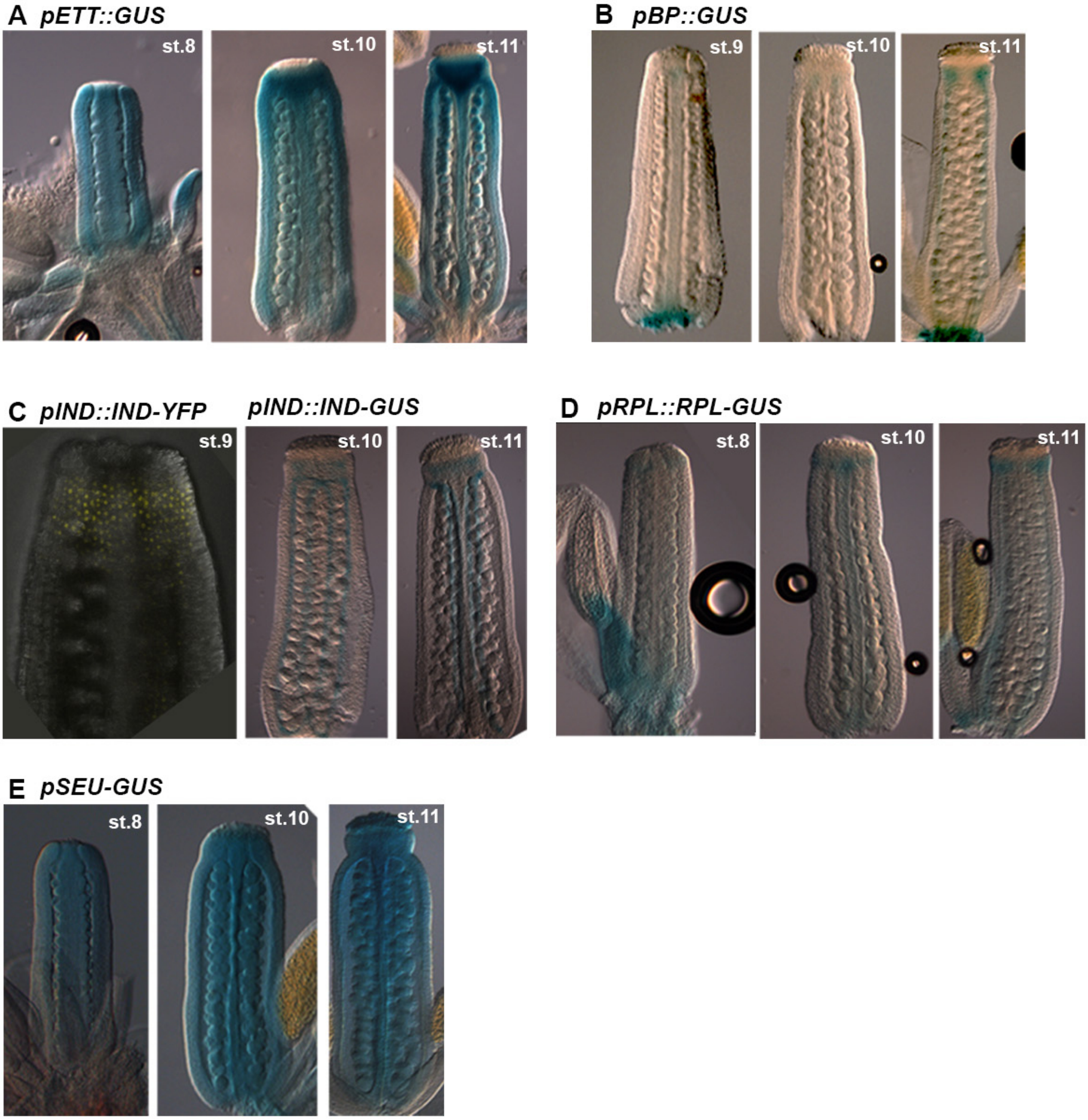

S1 Figure. Expression profile of ETT, IND, BP, RPL and SEU at different gynoecium developmental stages.
(A) Expression profile of pETT::GUS marker line at stages 8, 10 and 11 of gynoecium development.
(B) Expression profile of pBP:GUS marker line at stages 9, 10 and 11 of gynoecium development..
(C) Expression profile of pIND::IND-YFP (left panel, stage 9) and pIND::IND-GUS marker line (rigth, stages 10 and 11) at stages 9, 10 and 11 of gynoecium development.
(D) Expression profile of pRPL:RPL-GUS marker line at stages 8, 10 and 11 of gynoecium development..
(E) Expression profile of pSEU::GUS marker line at stages 8, 10 and 11 of gynoecium development.

Development • Supplementary information

S2 Figure.

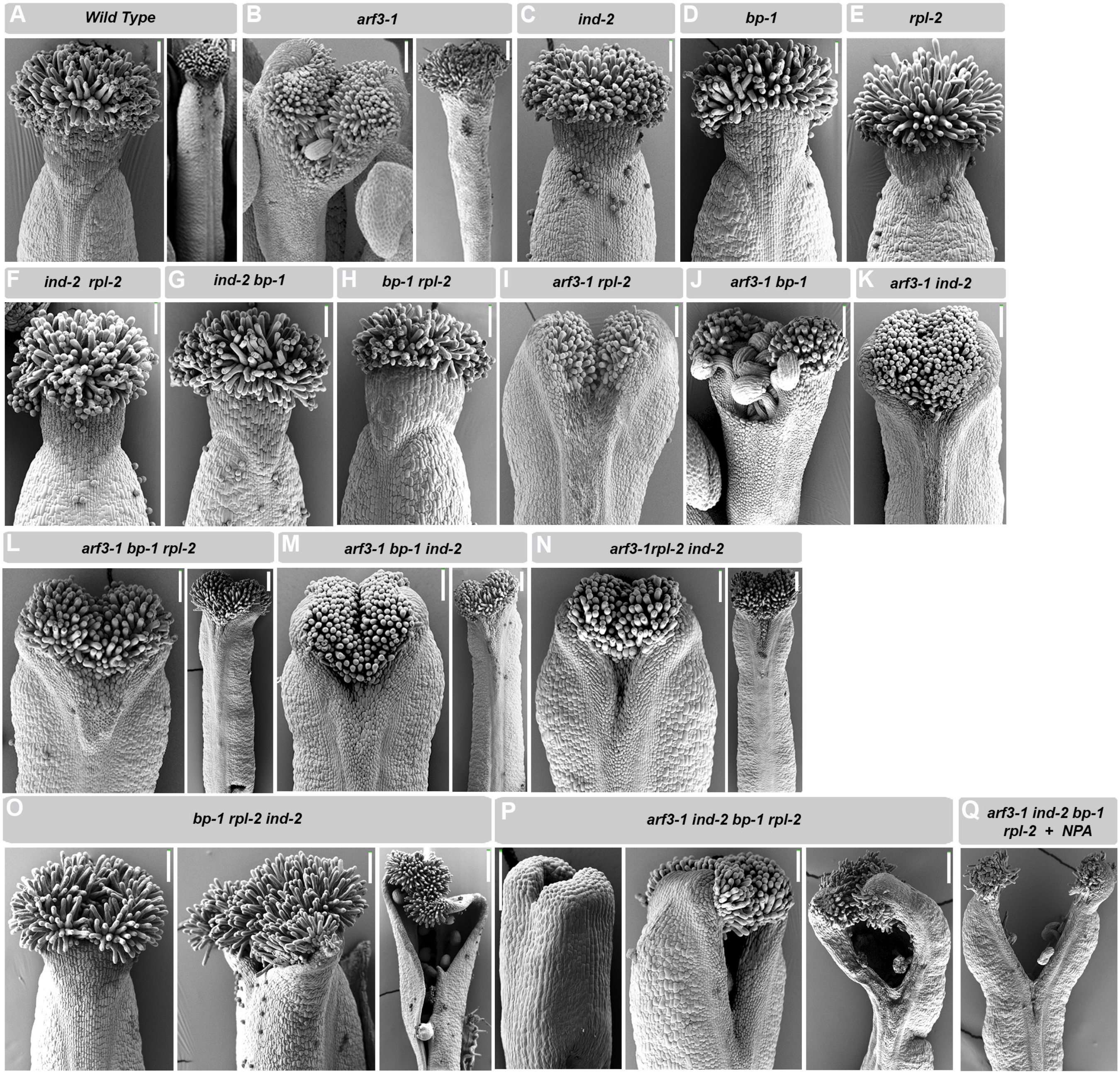

**R**	% of splitted gynoecia	% split length (tot gynoecium length = 100%)
**Single mutants**		
arf3-1	100%	20%
ind-2; *bp-1*; *rpl-2*	0%	0%
**Double mutants**		
arf3-1 ind-2; arf3-1 bp-1; arf3-1 rpl-2	100%	20%
ind-2 bp-1; ind-2 rpl-2; bp-1 rpl-2	0%	0%
**Triple mutants**		
arf3-1 ind-2 bp-1; arf3-1 ind-2 rpl-2; arf3-1 bp-1 rpl-2	100%	100%
bp-1 ind-2 rpl-2	11.2%	variable: from 10 to 40%
**Quadruple mutant**		
arf3-1 bp-1 ind-2 rpl-2	100%	variable: from 30 to 50%

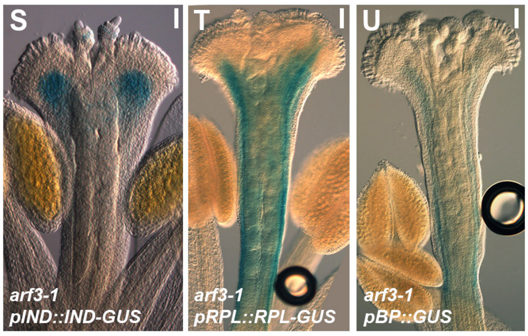

S2 Fig. Genetic interactions between ETT, BP, RPL and IND
(A-E) SEM images of wild-type (A), *arf3-1* (B), *ind-2* (C), *bp-1* (D) and *rpl-2* (E) single mutants.
(F-K) SEM images of *ind-2 rpl-2* (F), *ind-2 bp-1* (G), *bp-1 rpl-2* (H), *arf3-1 rpl-2* (I), *arf3-1 bp-1* (J) and *arf3-1 ind-2* (K) double mutants.
(L-O) SEM images of *arf3-1 bp-1 rpl-2* (L), *arf3-1 bp-1 ind-2* (M), *arf3-1 rpl-2 ind-2* (N) and *bp-1 rpl-2 ind-2* triple mutant.
(P-Q) SEM images of *arf3-1 bp-1 rpl-2 ind-2* quadruple mutant before (P) and after (O) NPA treatment.
(R) Percentage of splitted gynoecia and split length in the single, double, triple and quadruple mutant combinations.
(S-U) GUS staining of *pIND::IND-GUS* (R), *pRPL::RPL-GUS* (S) and *pBP::GUS* (T) in *arf3-1* mutant background.
Scale bar: 100µm

S3 Figure.

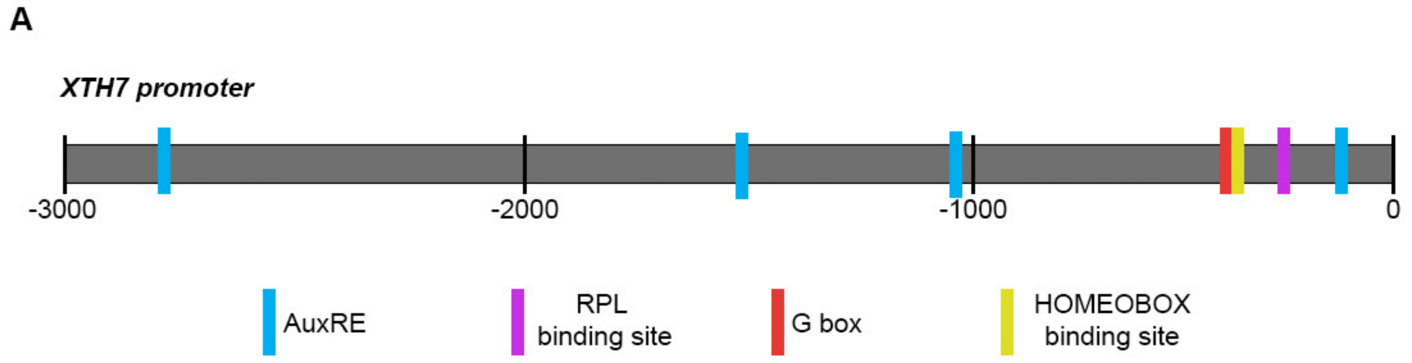

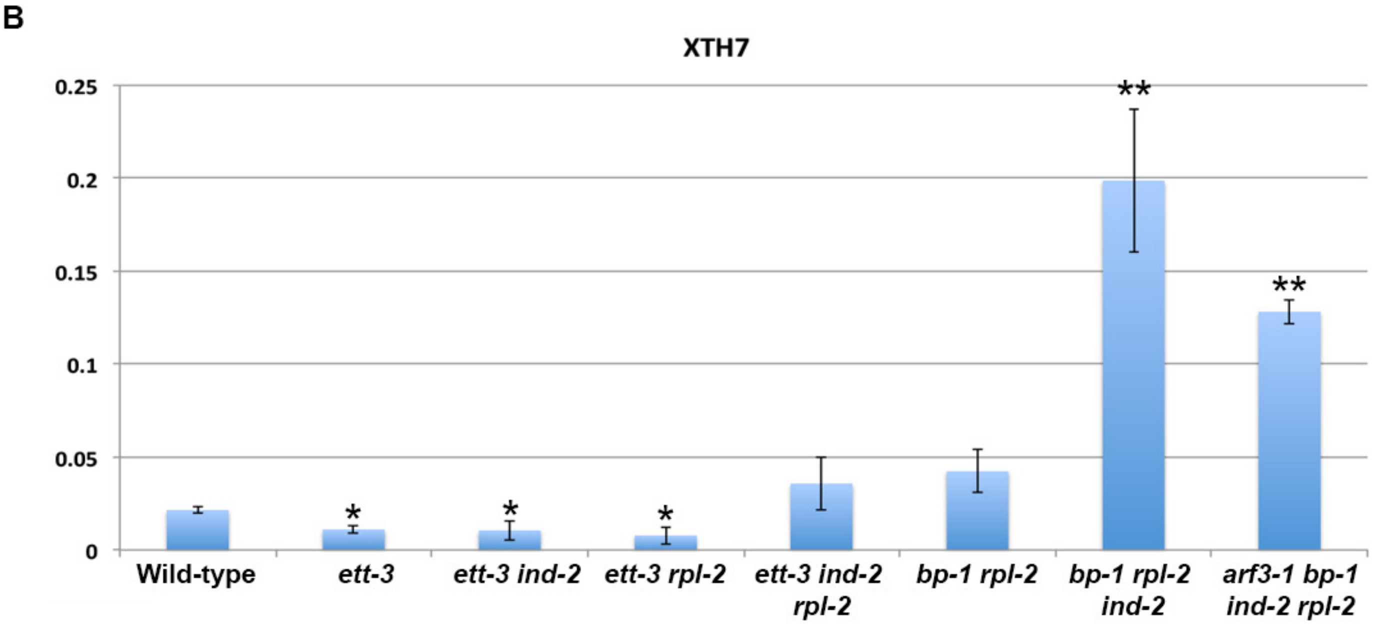

Figure S3. Expression analysis of *XTH7*.
(A) Schematic representation of potential binding sites for ARFs (AuxRE; ETT), bHLH (G box; IND), RPL and HOMEOBOX (BP) factors contained in the *XTH7* promoter.
(B) Expression analysis by quantitative PCR of *XTH7* in the indicated mutant combinations.
* is $p<0.05$; ** is $p<0.01$

Development • Supplementary information

S4 Figure.

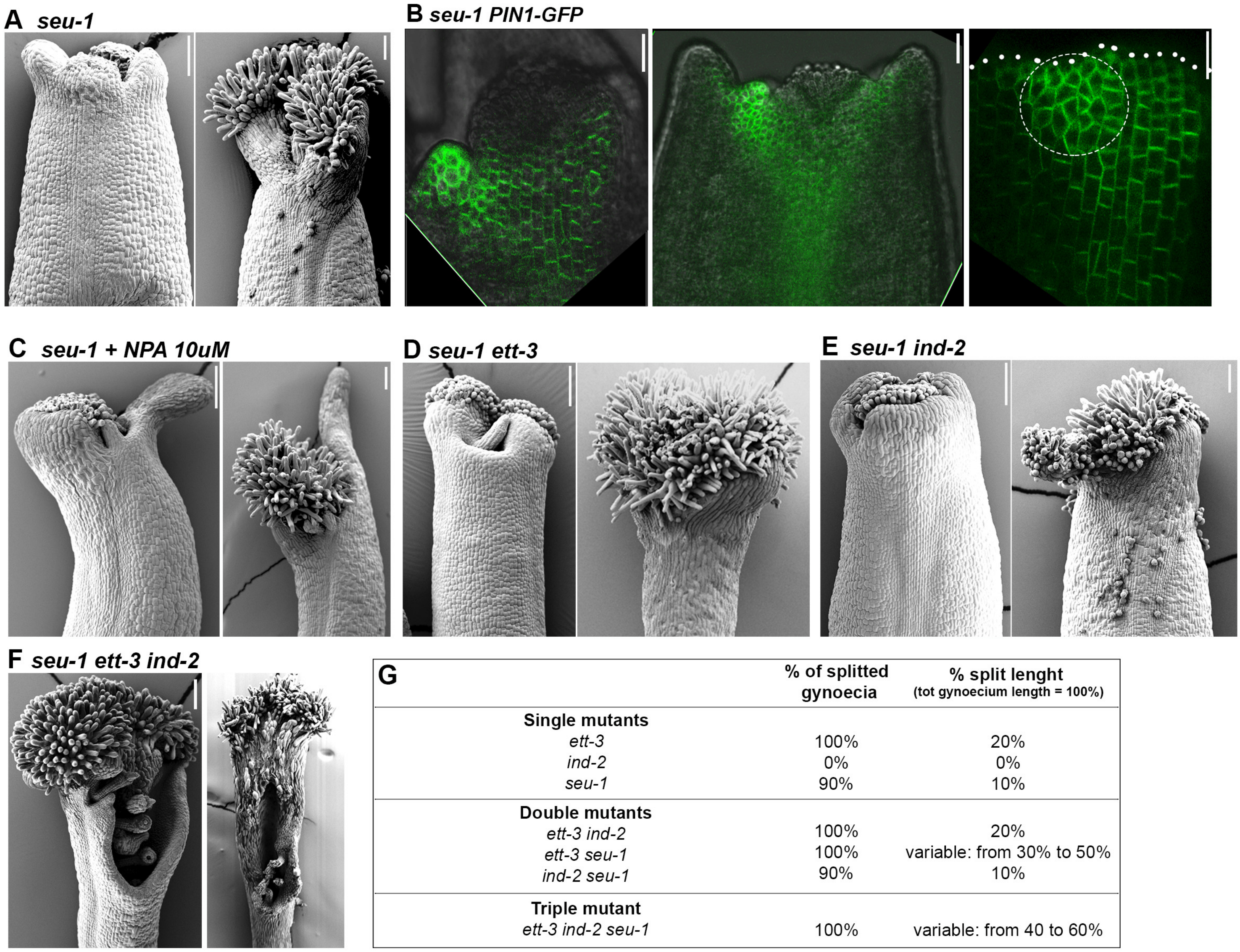

G

	% of splitted gynoecia	% split lenght (tot gynoecium length = 100%)
**Single mutants**		
ett-3	100%	20%
ind-2	0%	0%
seu-1	90%	10%
**Double mutants**		
ett-3 ind-2	100%	20%
ett-3 seu-1	100%	variable: from 30% to 50%
ind-2 seu-1	90%	10%
**Triple mutant**		
ett-3 ind-2 seu-1	100%	variable: from 40 to 60%

S4 Fig. Genetic interaction between ETT, IND and SEU and PIN1-GFP expression in *seu-1* mutant.
(A) SEM images of gynoecium apices of *seu-1* mutant.
(B) Confocal images of *pPIN1::PIN1-GFP* in *seu-1* mutant with focus on the top part of the gynoecium. Areas of PIN1 apical versus apolar localization can be seen adjacent to the developing cleft. Dotted circle in the third panel encircles an area where the cells present severely affected division plane.
(C-F) SEM images of gynoecium apices of *seu-1* mutant NPA-treated (C), *seu-1 ett-3* (D), *seu-1 ind-2* (E), *seu-1 ett-3 ind-2* triple mutant (F).
(G) Percentage of splitted gynoecia and lenght of the split in the single, double and triple mutant combinations.
Scale bar: 100µm

Development • Supplementary information

S5 Figure.

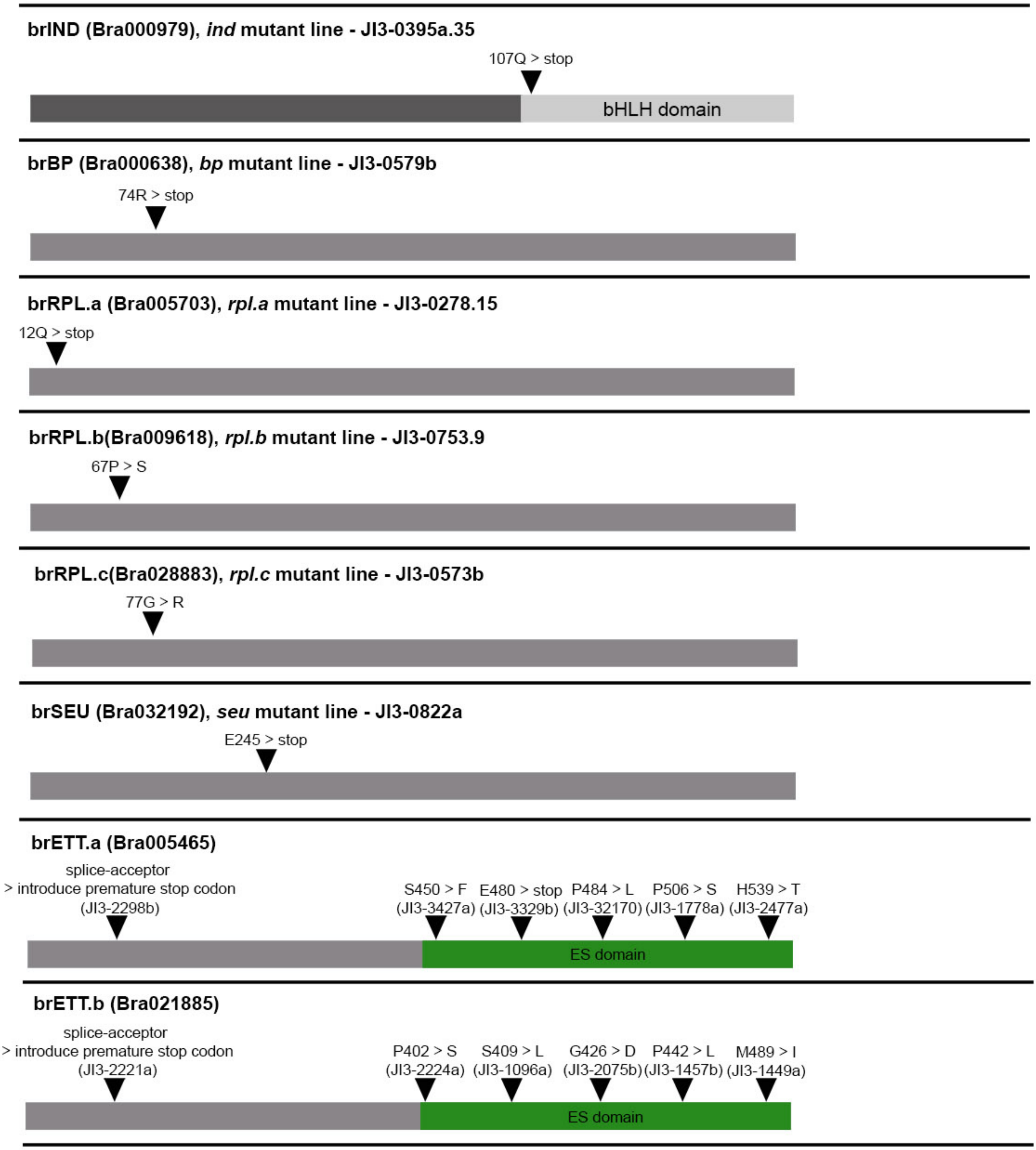

S5 Figure. **Schematic representation of Brassica rapa exome capture mutant lines analysed in this manuscript.**

Development • Supplementary information

S6 Figure.

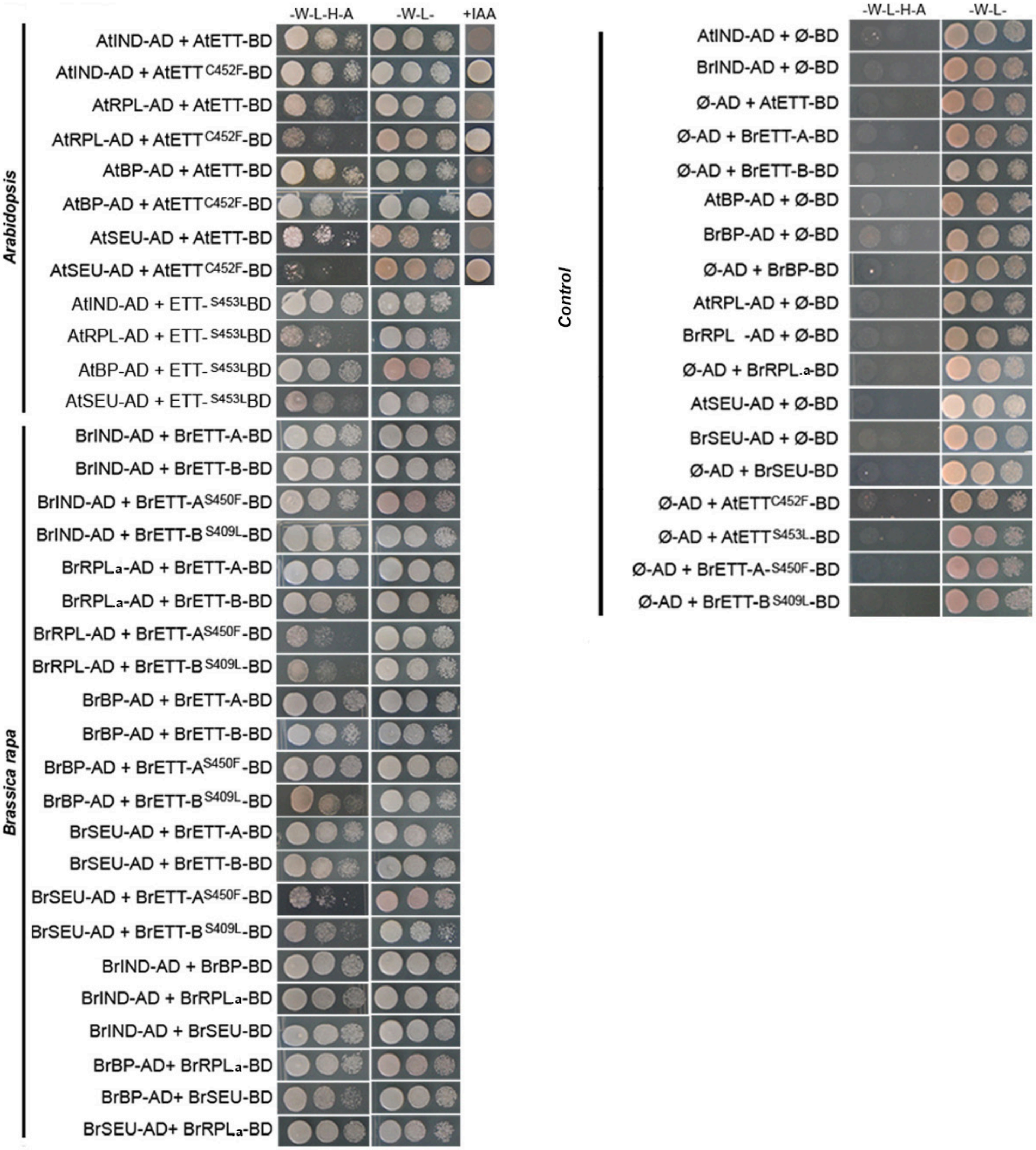

S6 Figure. Y2H combinations.

Y2H analyses between IND, ETT, RPL, BP and SEU proteins from *Arabidopsis* and *Brassica rapa* and respective controls.

Development 145: doi:10.1242/dev.158105: Supplementary information

S7 Figure.

A BrETT.a

MGGLIDLNVTEEQEEETTPSSGSGSLSPCDSSSSASAFGVSGSSSSSGVCLELWHACAGPLISLPKRGSLVLYFPQGHLEQAPDFSAAIYGLPPHVFCRILDVKLHAETATDEVYAQVSLLPES
EDVERKVREGVIDVDGGEEDYEVVKRTNTPHMFCKTLTASDTSTHGGFSVPRRAAEDCFPPLDYTQPRPSQELLARDLHGLEWRFRHIYRGQPRRHLLTTGWSAFVNKKKLVSGDAVLFLR
GDDGKLRLGVRRASQIEGASAFSSQYNQNMNHNNFAEVVHAISTNSAFNIYYNPKASWSNFIIPAPKFLKTVDYPFCIGMRFKARVESEDASERRSPGIITGINDLDPIRWPGSKWRCLLVRWD
DTDANGHHQQRISPWEIEPSGSISSSGSFITTGPKRSRIGFSSGKPDIPVSGSGATDFEESLRFQRVLQGQEIFHGFINTSSDGGAGARRGRFKGTEFGDSYGFHKVLQGQETVPAYPMMTDQ
RHQGMSQRNIWCGPFQNFSTRILPPSSVQVSSPLPSSGPNVRMEEHHGGSGRCRLFGFPLRDETTAVGSGMVVGPCVEGQNGSSIRGGVSAVQSSHHGRDIYGMRDMLLDIAL*

B BrETT.b

MGGLIDLNVTEEEDETLSSASGSLSTSNSSSSAFVANGSSSSSSSSGVCLELWHACAGPLISLPKRGSLVLYFPQGHLEQQAPGFSAAIYGLPPHVFCRILDVKLHAETDTDEVYAQVSLLPES
EDIERKLREGVIDVVDGGDQEDYEVVKRSNTPHMFCKTLTASDTSTHGGFSVPRRAAEDCFPPLDYSKPRPSQELLARDLHGLEWRFRHIYRGQPRRHLLTTGWSGFVNKKKLVSGDAVLFL
RGDDGKLRLGVRRASQFEGASAFSAQYNQNTNHNNFSEVAHAISTNSAFNIYYNPKSLGIITGISDLDPIRWPGSKWRCLIVRWDDNEANRHQQRVSPWEIEPSGSISSSGSFMTTGPKRSR
IGFPDIPVSEGIHATDFEESLRFQRVLQGQEKFPGFINTSSDGGAGARRGRFKGTEFGDSYGFHKVLQGQETVPAYSMTAHQQHGLSQGNFSSRFLPPSLVHVSSSPSSVLITNSNRPNGRL
EEHQGGSGSRCRLFGFPLRDESTAMVVPCVEGQKGVSGVQSNHHSQGRGIYGMRDMLLDIAL*

C

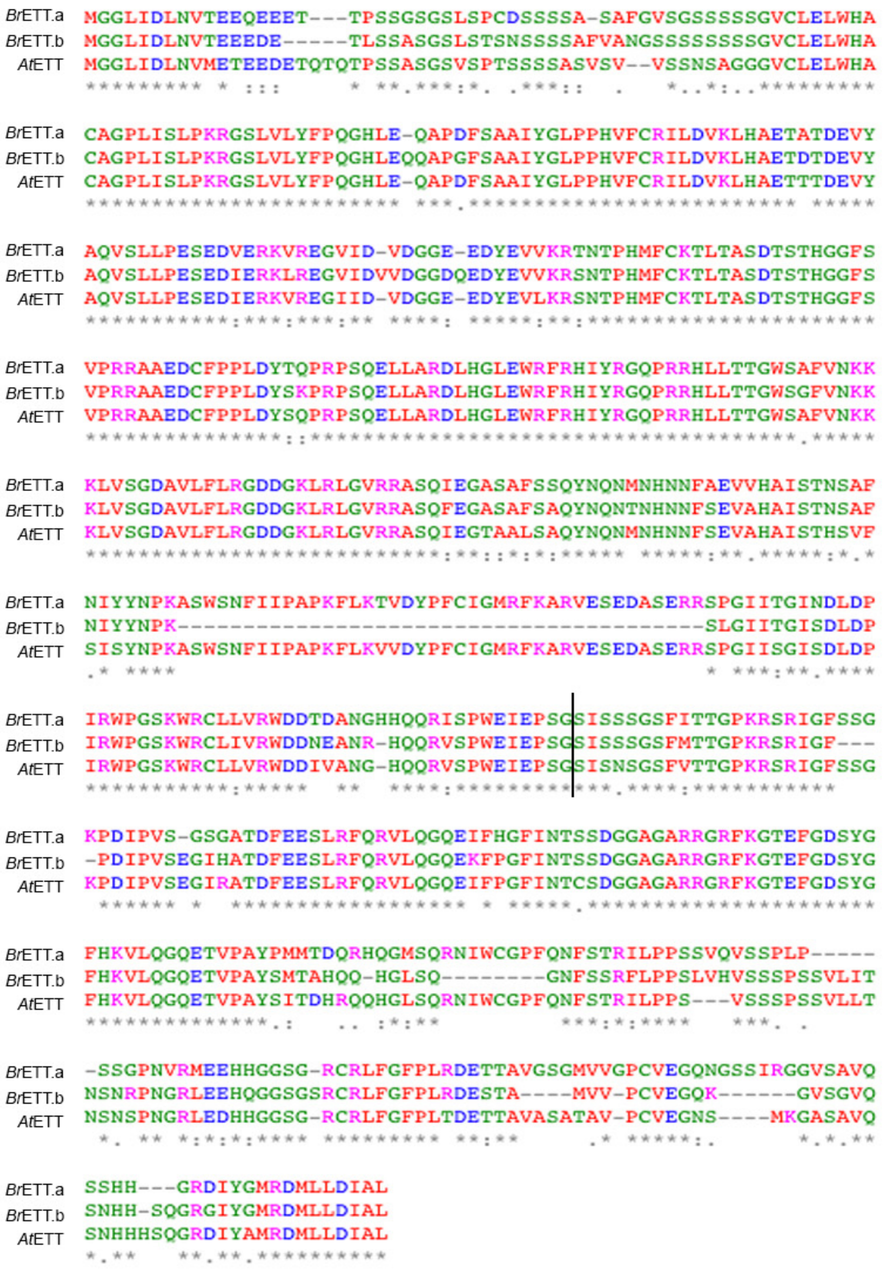

S7 Figure. ETT Protein alignment.

(A-B) Sequences of ETT proteins from B. rapa BrETT.a (A) and BrETT.b (B).
(C) Protein alignment between AtETT, BrETT.a, BrETT.b. Black vertical bar delimits the DBD domain (on the left) and the ES domain (on the right).

Development • Supplementary information

S8 Figure.

A ***ett-3 pETT::ETT-C452F***

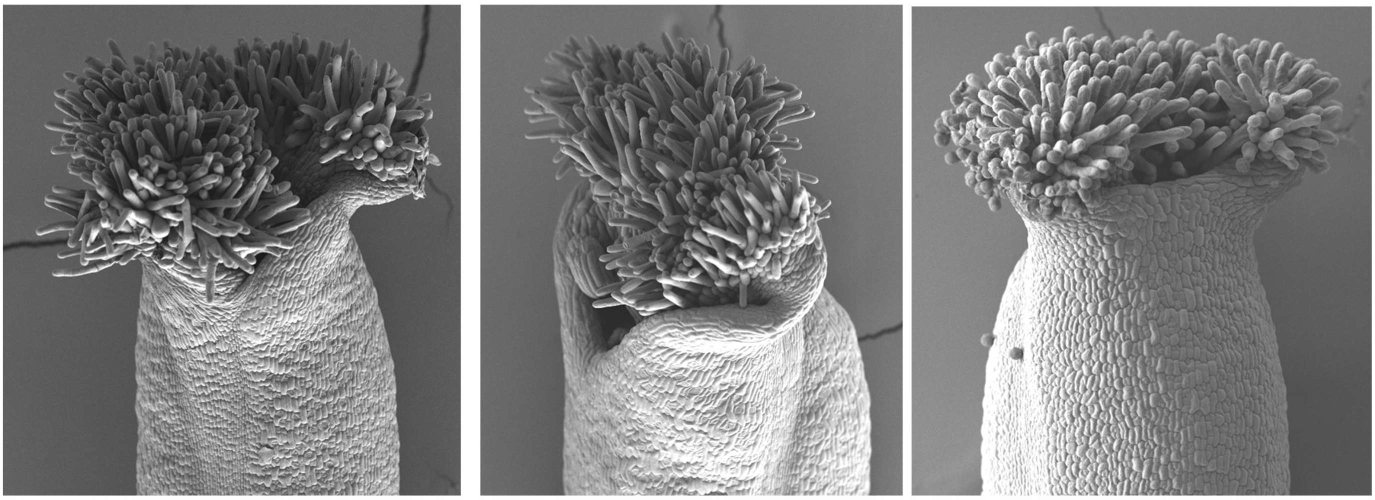

B

Amino acid	R	K	N	D	E	Q	H	P	Y	W	S	T	G	A	M	C	F	L	V	I
Kyte & Doolittle Hydrophobicity Scale	−4.5	−3.9	−3.5	−3.5	−3.5	−3.5	−3.2	−1.6	−1.3	−0.9	−0.8	−0.7	−0.4	+1.8	+1.9	+2.5	+2.8	+3.8	+4.2	+4.5
Physicochemical Meaning	More Hydrophilic													More Hydrophobic						

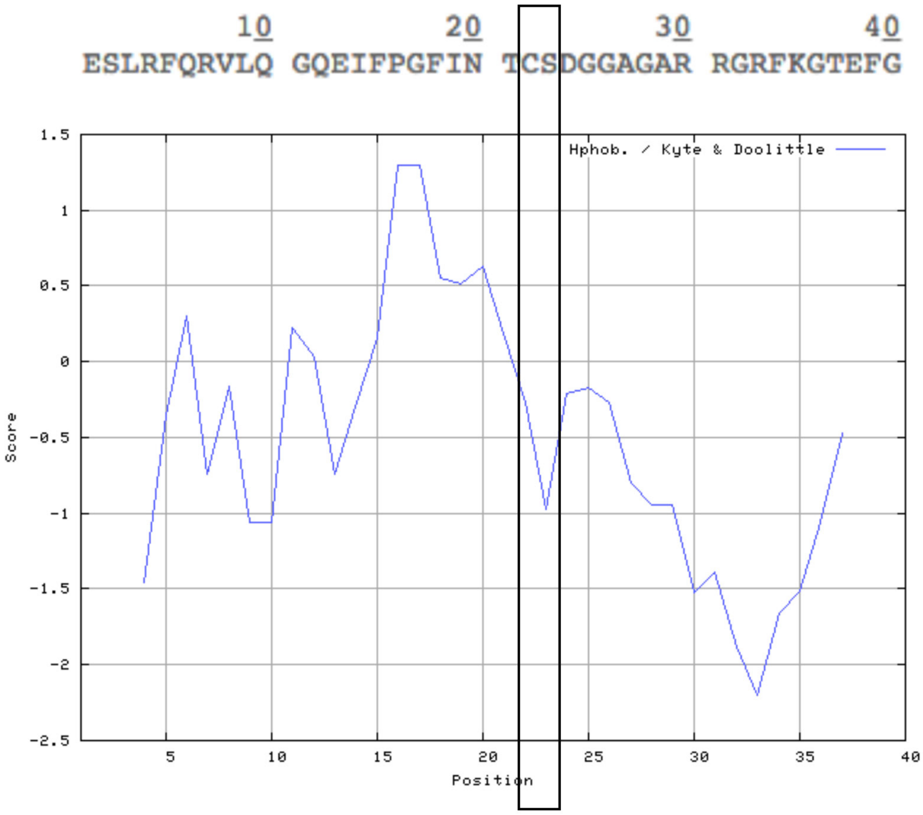

S8 Fig. Analyses of C452F substitution in AtETT
(A) SEM images of gynoecium apices of *ett-3* plants transformed with the pETT::ETT_C452F construct.
(B) Hydrophobicity analyses by ProtScale of the ETT subdomain where the C452F and S453L substitutions are located. The substitutions fall in a region which shows tendency to hydrophilicity with values below 0.

Development • Supplementary information
